# Elucidating the structure, and composition of bacterial symbionts in the gut regions of wood-feeding termite, *Coptotermes formosanus* and their functional profile towards lignocellulolytic systems

**DOI:** 10.3389/fmicb.2024.1395568

**Published:** 2024-05-22

**Authors:** Mudasir A. Dar, Rongrong Xie, Luohui Jing, Xu Qing, Shehbaz Ali, Radhakrishna S. Pandit, Chaitali M. Shaha, Jianzhong Sun

**Affiliations:** ^1^School of the Environment and Safety Engineering, Biofuels Institute, Jiangsu University, Zhenjiang, China; ^2^Department of Zoology, Savitribai Phule Pune University, Pune, India

**Keywords:** termites, gut-regions, bacterial diversity, microhabitats, high-throughput sequencing, symbiotic functions

## Abstract

The wood-feeding termite, *Coptotermes formosanus*, presents an efficient lignocellulolytic system, offering a distinctive model for the exploration of host-microbial symbiosis towards lignocellulose degradation. Despite decades of investigation, understanding the diversity, community structure, and functional profiles of bacterial symbionts within specific gut regions, particularly the foregut and midgut of *C. formosanus*, remains largely elusive. In light of this knowledge gap, our efforts focused on elucidating the diversity, community composition and functions of symbiotic bacteria inhabiting the foregut, midgut, and hindgut of *C. formosanus* via metagenomics. The termite harbored a diverse community of bacterial symbionts encompassing 352 genera and 26 known phyla, exhibiting an uneven distribution across gut regions. Notably, the hindgut displayed a higher relative abundance of phyla such as Bacteroidetes (56.9%) and Spirochetes (23.3%). In contrast, the foregut and midgut were predominantly occupied by Proteobacteria (28.9%) and Firmicutes (21.2%) after Bacteroidetes. The foregut harbored unique phyla like Candidate phylum_TM6 and Armatimonadetes. At the family level, Porphyromonadaceae (28.1, 40.6, and 53.5% abundance in foregut, midgut, and hindgut, respectively) and Spirochaetaceae (foregut = 9%, midgut = 16%, hindgut = 21.6%) emerged as dominant families in the termite’s gut regions. Enriched operational taxonomic units (OTUs) were most abundant in the foregut (28), followed by the hindgut (14), while the midgut exhibited enrichment of only two OTUs. Furthermore, the functional analyses revealed distinct influences of bacterial symbionts on various metabolic pathways, particularly carbohydrate and energy metabolisms of the host. Overall, these results underscore significant variations in the structure of the bacterial community among different gut regions of *C. formosanus*, suggesting unique functional roles of specific bacteria, thereby inspiring further investigations to resolve the crosstalk between host and microbiomes in individual gut-regions of the termite.

## Graphical abstract

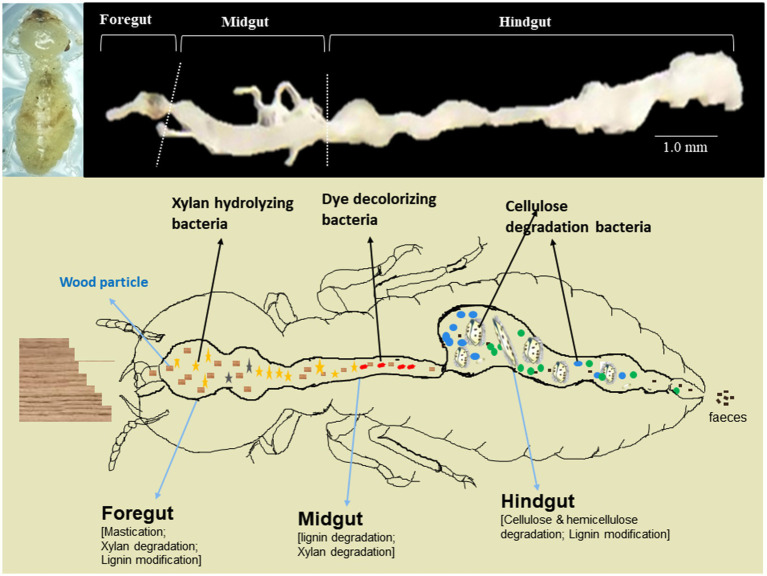

## 1 Introduction

Termites are one of the most successful insect lineages, that have evolved unique evolutionary adaptations linked to their eusocial lifestyle (Marynowska et al., 2020; Dar et al., 2022; Xie et al., 2023). Among termites, the invasive Formosan species, particularly from the genus *Coptotermes* demonstrate significant ecological and economic impacts globally due to their efficient enzymatic arsenal for lignocellulose metabolism (Husseneder et al., 2010). These wood-feeding species can digest over 90% of cellulose and 76% of hemicellulose within 24 h (Sun et al., 2014), posing threat to various plants (over 50 species), wooden structures, and buildings. The Formosan termites also known as super-termites exhibit destructive behavior, colossal colony sizes, and rapid digestion of wood cellulose (Lee et al., 2021), making them unique evolutionary models for efficient lignocellulose bioconversion (Brune, 2014). To achieve this expertise, termites have evolved a delicate yet highly efficient gut system (Xie et al., 2014; Geng et al., 2018a,b), marked by complex symbiotic mechanisms with gut microbiota including bacteria and flagellates (Brune, 2014). The gut system of termites is a long-elongated tube broadly divisible into foregut, midgut and hindgut regions. The foregut comprises esophagus, crop and gizzard while the midgut is a simple, slender-shaped tube distally marked by malpighian tubules. Foregut and midgut are relatively small in size, whereas hindgut is mostly enlarged, paunch that houses the bulk of the flagellate symbionts (characteristic feature of the lower termites). These digestomes are complex, microoxic fermenters and structured microenvironments with fundamental differences in their physicochemical conditions as well as other biotic and abiotic features (Brune, 2014). Many of the environmental features are intrinsic to the gut, while others result from the physiological collaborations with symbiotic bacteria, and flagellates residing in respective locations (Brune, 2014). Hitherto, several investigations have stated that symbiotic bacteria complement the host for maximum digestion of lignocellulosic biomass, contributing to an impressive enzyme system for extracting carbohydrate energy from wood (Sun et al., 2014). Beyond digestive symbiosis, gut bacteria are integral to termite eusociality, immunity, and nitrogen metabolism (Engel and Moran, 2013). Furthermore, some bacterial symbionts possess the inherent ability to degrade aromatic hydrocarbons, adding to the multifunctionality of termites (Sun et al., 2014). Thus, deciphering the diversity, composition, and functional profiles of bacterial symbionts in individual gut-regions becomes immensely important to underscore the physiology and evolution of termites.

During the last two decades, there has been a notable surge in molecular ecological studies utilizing ‘omics’ technologies particularly metagenomics, to unravel the structure of bacterial communities in termite guts (Hongoh, 2011). The high-throughput sequencing like metagenomics has successfully resolved key differences in microbiota community structures among different termite species (Scharf, 2015a). Lazuka et al. (2018) reported on the community dynamics of anaerobic bacteria with characteristic xylanase activities in a higher termite, *Nasutitermes ephratae*. Similarly, Warnecke and colleagues elucidated the bacterial community in the hindgut paunch of *Nasutitermes* sp. by using metagenomics (Warnecke et al., 2007). Although omics technologies and integrative systems biology approaches have substantially advanced our understanding of termite biology, particularly their eusociality, pathogen defense, and digestive symbiosis, still many knowledge gaps remain unexplored. In particular, the gut-region-specific diversity and interaction of bacterial phylotypes with host termite for lignocellulose digestion is largely elusive. Furthermore, the functions of bacterial symbionts residing in the foregut and midgut are poorly studied, likely due to the smaller size (10–20 μL) of these compartments and associated technical challenges (Tokuda et al., 2005). Unlike the hindgut where flagellates are recognized for cellulose digestion (Brennan et al., 2004), the fate of lignocellulose in the foregut and midgut that are devoid of protozoan flagellates, is still unknown. In addition, the majority of the studies are focused on higher termites, characterized by a simpler two-way association between host and bacterial endosymbionts (Scharf, 2015b). However, in lower termites, the presence of cytoplasmic and intranuclear endosymbionts in flagellate cells (e.g., Elusimicrobia in *Reticulitermes* sp.) adds to the complexity of the process (Stingl and Radek, 2005), challenging to revelation of individual microbiota contributions and interactions. Consequently, limited information exists regarding bacterial diversity and community structures in different gut-regions of the lower wood-feeding termite, *Coptotermes formosanus*. Further, the functional profiles of bacteria elucidating interactions between host and symbionts during survival have not been thoroughly examined in *C. formosanus*.

The wood-feeding termite, *C. formosanus* Shiraki associates with gut symbionts to digest over 90% of cellulose and ~ 60% hemicellulose (Xie et al., 2012). This Formosan subterranean termite widespread in distribution, serves as an important structural pest in temperate and subtropical regions causing substantial economic losses globally (Geng et al., 2018a; Lee et al., 2021). *Coptotermes formosanus* exhibits a broad dietary spectrum, consumes anything that contains wood fiber, such as homes, buildings, crops, plants, and live trees (Lee et al., 2021). A mature colony of *C. formosanus* having millions of individuals can swiftly consume large amounts of wood (~400 g per day), leading to severe damage to wooden structures within a short period. The annual global economic impact of subterranean termites, with *Coptotermes* as a major contributor is estimated at USD$32 billion (Rust and Su, 2012). The *C. formosanus* has been reported as the most destructive termite in southern China (Wang et al., 2002), USA (Su, 2003), Japan (Lax and Osbrink, 2003), Malaysia (Lee, 2002), etc. causing tremendous damage to wooden infrastructures. Recently we observed that *C. formosanus* achieves maximum lignocellulose digestion through symbiosis with gut microbiota particularly bacteria (Geng et al., 2018b; Dar et al., 2022). Additionally, the metatranscriptomic profile of its flagellates also revealed the contribution of lignocellulase encoding bacterial genes, further highlighting the complex processes involved in lignocellulose breakdown (Xie et al., 2012). Despite the reported existence of bacterial symbionts in *C. formosanus* (Xie et al., 2012; Dar et al., 2022), our understanding of the specific processes occurring within individual gut-regions of this termite is fragmentary. Therefore, further research is imperative to elucidate the intricate interplay between gut microbiota and the host toward lignocellulolytic systems.

A comprehensive investigation into the bacterial compositions of individual gut-regions would delineate the predominant lineages that shape the structure of bacterial communities in termites (Mikaelyan et al., 2017). Elucidating the region-wise structure and composition of the bacterial communities in termites will improve our understanding of the functional crosstalk between symbiotic microbiota and the host during the programmatic digestion of lignocellulose. In this context, the comparative structure and functional profile of bacterial symbionts residing in different gut-regions of *C. formosanus* warrants thorough investigation. Therefore, we attempted to address this lacuna by fractionating the diversity and community structure of bacteria residing in the individual gut-regions, such as foregut, midgut, and hindgut of *C. formosanus* by using metagenomic analyses. Additionally, we elucidated the potential functions of these bacteria in lignocellulose digestion and other metabolic processes within each gut-region of the termite. Our results provide significant insights into microbial ecology uniquely existing in hitherto unexplored foregut and midgut of *C. formosanus*. The present study further elaborates our knowledge of termite biology that might help to design an efficient bioreactor in lignocellulose degradation via biomimetics besides its significance for integrated pest management.

## 2 Materials and methods

### 2.1 Chemicals and reagents

The HiPure Soil DNA Kits were purchased from Magen biotechnology (Magen Pvt. Ltd. China). The Kod Plus buffer and DNA polymerase were purchased from TOYOBO (Japan) along with, other reagents required for PCR amplifications. The AxyPrep^™^ gel DNA extraction kit and StepOnePlus^™^ Real-Time PCR system were procured from Axygen (Axygen Biosciences, CA, United States) and ABI Life Technologies (Life Foster City, United States) respectively.

### 2.2 Collection and dissection of the termites

The wood-feeding *C. formosanus* termites were collected from Shaoguan City (24°55′8″ N, 113°57′44″ E), Guangdong province, P. R. China. Since *C. formosanus* is among the 100 worst alien invasive species causing tremendous damage to wooden structures and forests, feeding predominantly on pine trees (Lee et al., 2021). We reared and maintained these termites in the laboratory at 26 ± 2° with R.H. ~60–80% by feeding *ad libitum* on pine wood, *Pinus massoniana* (Mikaelyan et al., 2015; Dar et al., 2022). To avoid the contamination from environmental microbes including bacteria, the pine wood blocks were autoclaved at 121°C for 15 min before feeding to the termites (Mitaka and Vargo, 2023). A total of 1,050 worker termites were carefully dissected in a biosafety hood using sterilized instruments (Dar et al., 2022). The termites used for the experimentation comprised three different groups that were reared from the same stock colony. Within each group, approximately 350 ± 20 worker termites were subjected to dissection to reveal the individual gut regions. Prior to dissection, the adult worker caste termites were surface sterilized with 50 and 70% ethanol in distilled water each for 30 s, followed by a brief wash of sterile double distilled water (SDDW). After dissection, the gut systems were divided into foregut, midgut- and hindgut (Supplementary Figure S1) with the aid of 30X portable optical loupe by using sharp and sterilized needles. Then, each of the gut-regions was separately placed in 1.5 mL micro-centrifuge tubes (MCT), frozen immediately in liquid nitrogen, and stored at −80°C till further use. The dissection and processing of the gut-regions were carried out carefully to eliminate the chances of bias caused due to the mixing of the gut contents. The gut-regions were suspended in 500 μL of sodium phosphate buffer solution (50 mM; pH 7.0) and subsequently homogenized with polypropylene micro-pestles. The experiments were replicated three times, with each replication involving the consideration of 350 ± 20 termites to retrieve the individual gut regions.

### 2.3 Microbial DNA extraction and Illumina sequencing

The microbial community DNA was extracted by using the HiPure Soil DNA extraction Kit (Magen, Guangzhou, China) following the manufacturer’s instructions. The extracted DNA was quantified and quality checked on a nanodrop Biospectrophotometer. The V3 and V4 regions of the 16S rDNA genes of the bacterial community were PCR amplified (Liu et al., 2019) by using the primers listed in Table 1. The PCR amplifications were operated at 94°C for 2 min, 30 cycles at 98°C for 10 s, 55°C for 30 s, and 68°C for 30 s followed by a final extension step at 68°C for 5 min. The PCR amplifications were performed in triplicates using a 50 μL reaction mixture comprising 5 μL of 2 mM dNTPs, 3 μL of MgSO_4_ (25 mM), 5 μL of 10× KOD Buffer, 1.5 μL of each primer (10 μM), and 1 μL of KOD Polymerase added to 100 ng of template DNA. The successful PCR amplicons were extracted from agarose gels (2%) and then purified by using an AxyPrep DNA Gel extraction kit (Axygen Biosciences, CA, United States) according to the manufacturer’s instructions followed by quantification with ABI StepOnePlus Real-Time PCR System (Life Technologies Foster City, United States). The quantified amplicons were pooled and used to construct paired-end DNA libraries. The constructed libraries were sequenced (250 paired-end sequencing) on the Illumina MiSeq platform according to the standard protocols.

**Table 1:** Primer sets used for the generation of metagenomic libraries based on target regions of 16S rDNA gene of the bacterial communities in *C. formosanus*.

Target gene	Target region	Primer pair	Nucleotide sequence	Product size	References
16S rDNA	V4	515F	5′-GTGYCAGCMGCCGCGGTAA-3′	~292	Parada et al. (2016), Apprill et al. (2015)
		806R	5′-GGACTACNVGGGTWTCTAAT-3′		
	V3-V4	341F	5′-CCTACGGGNGGCWGCAG-3′	~466	Guo et al. (2017)
		806R	5′-GGACTACNVGGGTWTCTAAT-3′		

### 2.4 Sequence data analysis

The obtained raw sequences were processed by using the Quantitative insights into microbial ecology software v1.9.1 (QIIME, Caporaso et al., 2010). Before analysis, the raw sequence reads containing adapters or low-quality reads were quality-filtered using FASTP software v0.18.0 (Chen et al., 2018). The quality clean-up was carried out by removing the sequence reads containing more than 10% unknown nucleotides and base quality (*Q*-value) > 20. Subsequently, the paired-end clean reads were merged into consensus sequences using FLASH v1.2.11 (Magoč and Salzberg, 2011) based on the overlaps longer than 10 bp and a mismatch error rate of 2%. All chimeric tags were removed by UCHIME algorithm v4.2 (Edgar et al., 2011), resulting in effective tags that were used for further analysis. These effective tags were clustered into operational taxonomic units (OTUs) based on ≥97% sequence similarity using UPARSE pipeline v9.2.64 (Edgar, 2013). The number of OTUs was summarized with USEARCH 7.0 to generate OTU data table for each group. The annotation of the OTUs was performed by the Ribosomal Database Project classifier (RDP), and then the representative sequences of each OTU were selected for taxonomic information with an identity threshold of 0.8 (Wang et al., 2007). The initial OTU matrix often contains several OTUs showing extremely low abundance that reduces the number of OTUs having high abundances thereby increasing the complexity of the data analysis. The elimination of these rare OTUs depicts a negligible effect on bacterial diversity, while significantly increasing the efficiency of data analysis. Therefore, OTUs that showed a relative abundance of less than 0.1% of all OTUs were excluded from the analysis (Bokulich et al., 2013). The rarefaction curves representing the bacterial diversity of each sample were drawn in the Mothur software package (Kemp and Aller, 2004) to determine the sequencing depth.

### 2.5 Comparative analysis of the community structure in *Coptotermes formosanus*

**Figure 1:**
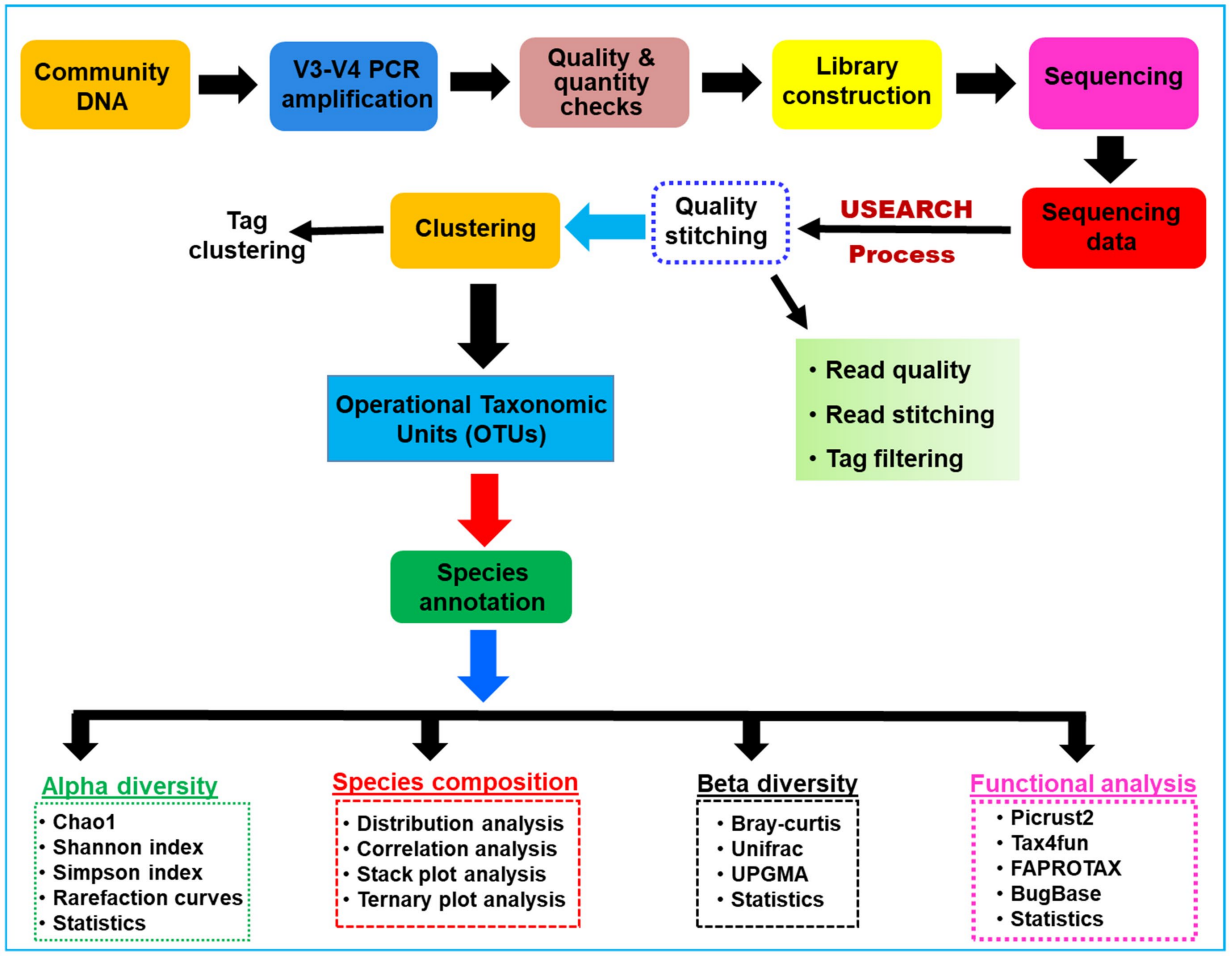
A schematic overview of the methodologies and algorithms used for the processing of the metagenomic data related to the gut microbial communities of *C. formosanus*.

The sampling depth of the obtained OTUs was estimated from rarefaction curves by using good’s coverage (Good, 1953). An overview of the work flow of methodology used for the present study is provided in Figure 1. The Alpha diversity index such as expected richness (Chao, 1984), diversity (Chao and Shen, 2003), and Evenness in terms of Chao, Shannon, and Simpson indices of the bacterial communities were calculated for each sample in QIIME (Caporaso et al., 2010). The community structure was compared between the gut regions by using the taxonomy-dependent Bray-Curtis metrics, a statistical test used to quantify the compositional dissimilarity among samples (Bray and Curtis, 1957). To visualize the dissimilarity among the gut-regions, the high dimensionality of the pairwise dissimilarity scores were compressed into two dimensions and plotted on non-metric multidimensional scaling (NMDS) using vegan package v2.5.3 (Oksanen et al., 2010) in R program. Similarly, the covariance between the community structure of gut compartments was determined by permutation multivariate analysis of variance (PERMANOVA) and subsequently visualized by principal coordinate analysis (PCoA) using the adonis function implemented in the vegan package (Ramette, 2007). Later, the biomarker features in each group were screened by LEfSe software version 1.0 (Segata et al., 2011), in the R project. Similarly, the ternary plots depicting the species abundance were plotted using R ggtern package v3.1.0 (Hamilton and Ferry, 2018). Further, the tests of significance for both intragroup and intergroup Unifrac distances were carried out by using the Monte Carlo permutation test in the QIIME software. To explore the differences between the intergroup distances, an analysis of similarity (ANOSIM) was performed and effects were visualized through biplots in the R project using the vegan package.

### 2.6 Functional prediction analysis of the bacteria

The functional characterization of bacterial communities residing in the gut system of animals is the key element to understanding host-symbiont interactions. To accomplish this, the KEGG pathway analysis of the OTUs was inferred by using Tax4Fun v1.0 (Aßhauer et al., 2015) by predicting the potential functions of observed microbial communities within the termite gut. Further, the microbiome phenotypes were classified using Bug Base (Ward et al., 2017). The functional annotation of the prokaryotic taxa database (FAPROTAX) v1.0 (Louca et al., 2016) was used for generating the ecological functional profiles of the gut bacteria. The analysis of function difference between bacterial groups was calculated by Tukey’s HSD test in the R project using the Vegan package (version 2.5.3, Oksanen et al., 2010).

### 2.7 Nucleotide sequence accession number

The sequences obtained in this study are deposited to the NCBI Sequence Read Archive database with Bioproject ID, PRJNA1013132. The accession numbers for the sequence reads are SRR25919998 to SRR25920006.

### 2.8 Data analyses

The results are presented as means and standard deviations derived from three replicates. The data were subjected to statistical analyses in R program v4.3.1 (R Core Team, 2023), and the principal component analysis were performed to generate the biplots of first two components by using ggtern package v3.1.0 (Hamilton and Ferry, 2018). A *p ≤* 0.05 was considered statistically significant. Each experiment was replicated at least three times to ensure the reproducibility of the results.

## 3 Results

### 3.1 Data set statistics

**Figure 2:**
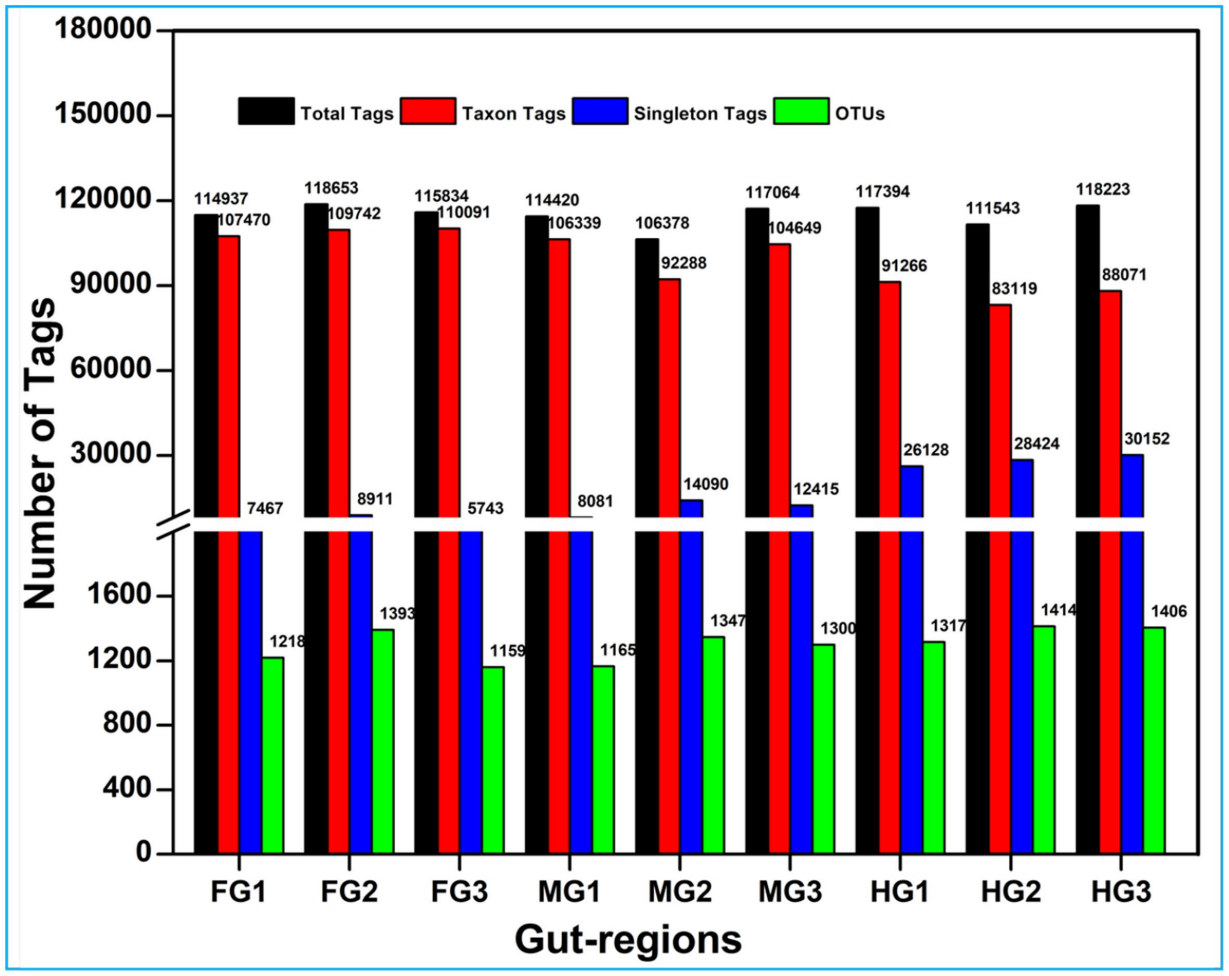
Summary of the overall processing of taxon tags for species annotation to operation taxonomic units (OTU).

A total of 1.17 million pair-end reads were obtained from 9 metagenomic libraries by sequencing the V3 and V4 regions of the bacterial communities, derived from the foregut, midgut, and hindgut of the wood-feeding termite, *C. formosanus*. These pair-end reads were processed into 1,172,293 clean-end reads. The high-quality cleaned reads were further binned and filtered into 1,072,995 raw tags (Table 2). These raw tags were further processed for chimera removal resulting in 1,034,446 effective tags. Subsequently, the effective tags were classified into 893,035 taxon tags after removing 141,411 singleton tags. The high-quality reads based on the read length of ~450 bp led to the assignment of 3,505 OTUs after defining 97% sequence similarity (Figure 2). The calculated rarefaction curves inferred from species richness reached a plateau, except for a few more diverse samples from hindgut (Supplementary Figure S2). However, the good’s coverage depicted efficient sequencing depth covering over >99.4% of the bacterial communities harbored by the termite. The presence of the Archaeal sequences was not detected in the microbial metagenomes. Since bacteria comprise an integral component of the termite gut microbiota, all of the divisions found in our metagenomic analysis were bacterial lineages, suggesting their indispensable role in fostering digestive symbiosis among termites.

**Table 2:** Summary statistics of the annotation of tags generated from the bacterial metagenomes in the gut system of *C. formosanus*.

Sample	Domain	Phylum	Class	Order	Family	Genus
FG1	107,470	106,541	106,277	104,800	102,231	93,432
FG2	109,742	109,544	109,277	108,011	98,591	74,105
FG3	110,091	109,835	109,457	108,913	104,244	84,462
MG1	106,339	105,465	105,065	103,904	101,878	94,717
MG2	92,288	91,854	91,370	90,841	88,200	75,961
MG3	104,649	104,048	103,848	103,619	102,474	95,385
HG1	91,266	90,530	89,689	88,519	87,372	78,971
HG2	88,071	87,380	86,513	82,360	81,582	69,686
HG3	83,119	82,422	81,576	79,621	78,756	69,842
Total	893,035	887,619	883,072	870,588	845,368	736,561

The sample codes represent three replicates for each of the foregut, midgut and hindgut metagenomes.

### 3.2 Bacterial complexity of the microbiome in *Coptotermes formosanus*

**Figure 3:**
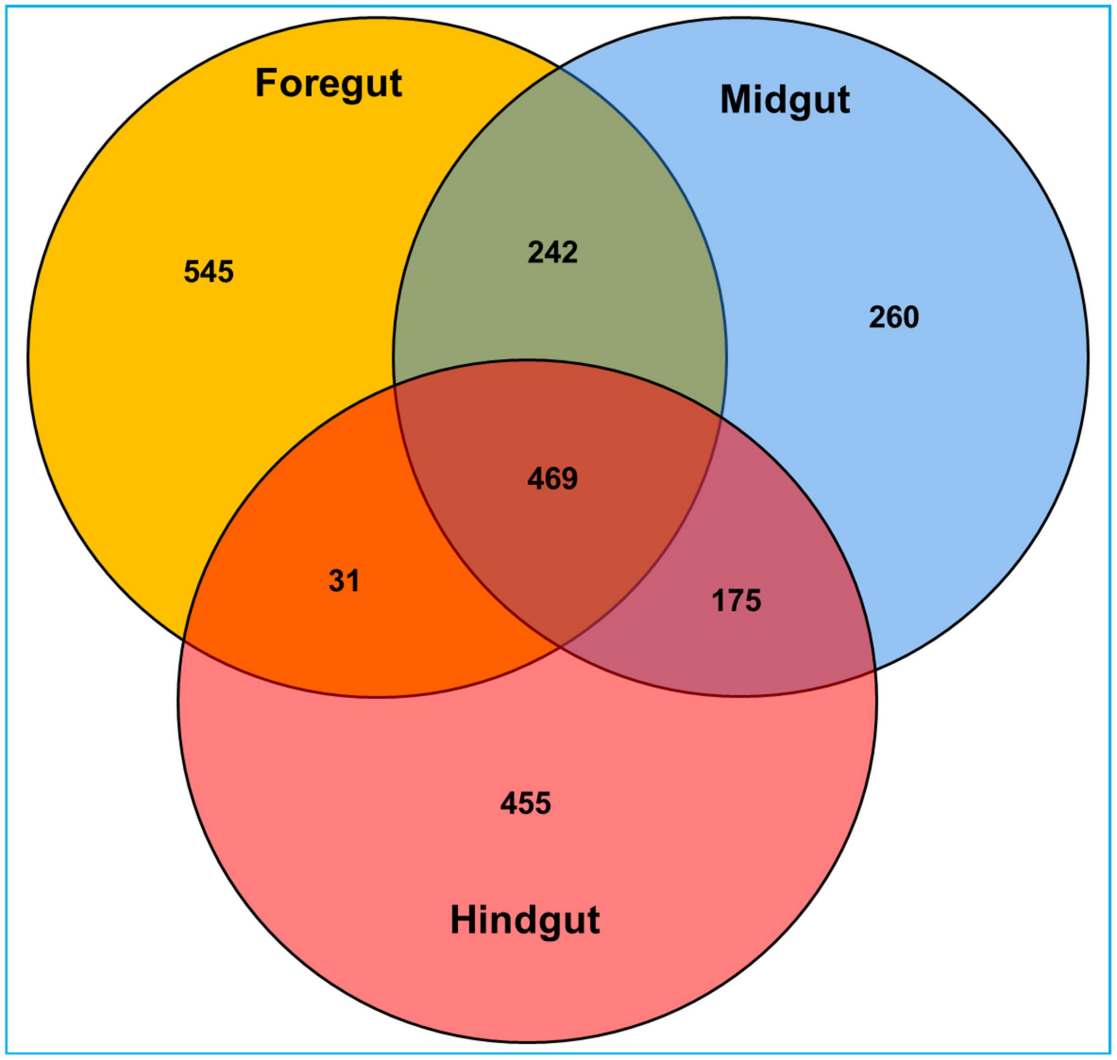
Venn diagram depicting the number of unique and shared operational taxonomic units (OTUs) between the gut-regions of wood-feeding lower termite, *C. formosanus*.

Among the gut regions, the highest number of taxon tags were achieved with foregut (109101) followed by midgut and hindgut which contributed 101,092 and 87485.3 taxon reads, respectively. Of the 3,505 OTUs observed in the gut system of *C. formosanus*, the indicator species analysis revealed an abundance of 2,177 OTUs only, among the three gut-regions of the termite. Among them, the maximum number of OTUs was harbored by foregut having 1,287 OTUs. Though lower than the foregut, the midgut and hindgut regions sheltered an average of 1,146 and 1,130 OTUs, respectively (Figure 2). Approximately 500 OTUs assigned to species-level classification were shared between the foregut and hindgut. The highest number of 711 OTUs were shared between the foregut and midgut regions. The total OTUs shared by the midgut with hindgut was found to be 644 while 469 OTUs were shared between all the three gut-regions under consideration. The number of unique OTUs were observed as 545, and 455 for foregut, and hindgut respectively, while being lowest for the midgut which restricted the unique OTUs to 260 only (Figure 3).

**Figure 4:**
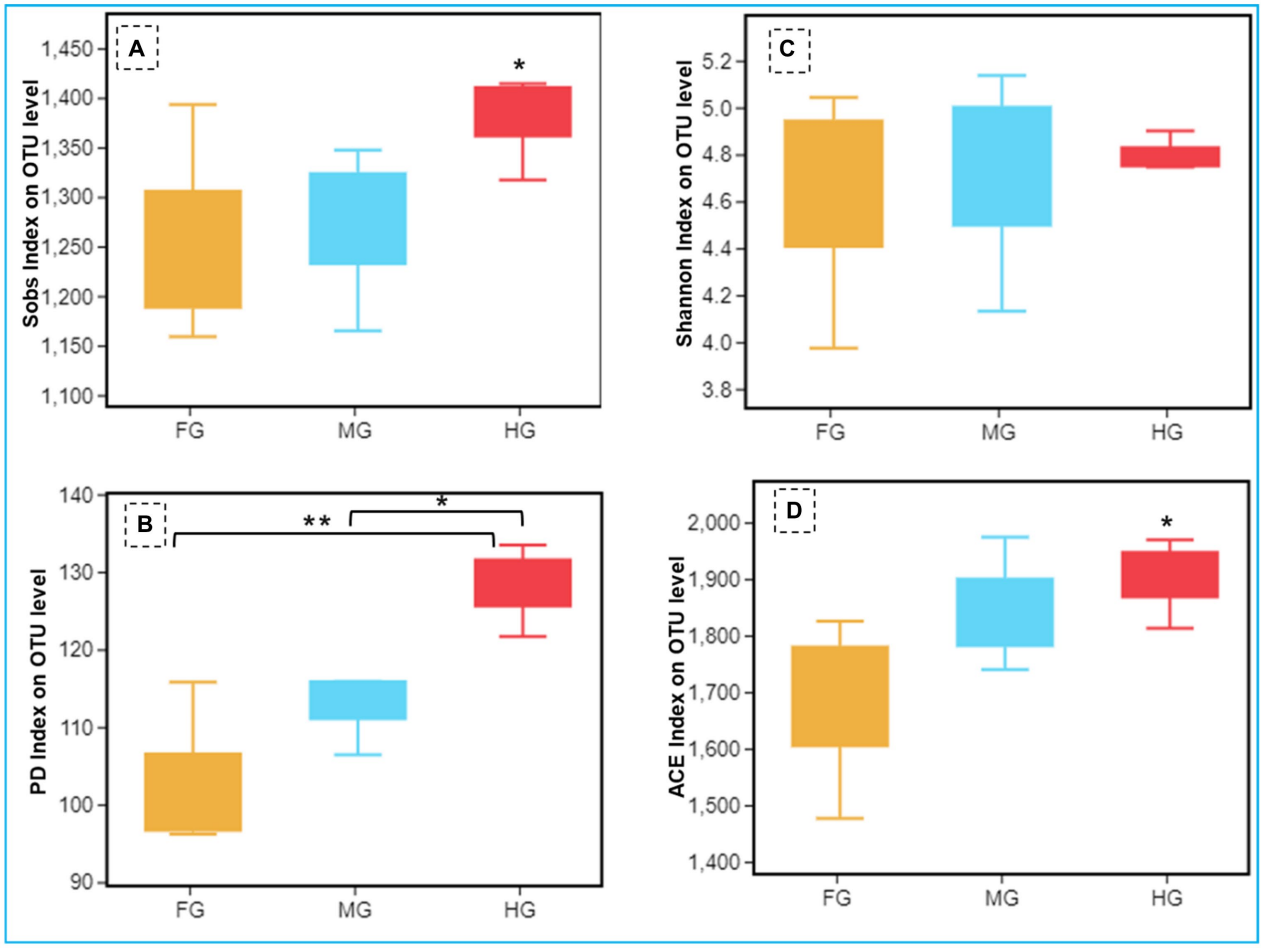
Comparison of alpha diversity (**A**: Sob index, **B**: Shannon index, **C**: phylogenetic diversity index, and **D**: ACE index) of the bacterial communities of *C. formosanus* between Foregut, midgut and hindgut regions (Tukey’s HSD test, *p ≤ 0.05 and **p ≤ 0.01).

The Shannon index of 5.48 together with Simpson and Pielou indices (0.87 and 0.54 respectively) suggested higher bacterial diversity in the foregut than midgut and hindgut. However, the nonparametric estimation of the bacterial species demonstrated highest species richness in hindgut depicting a chao1 value of 1,722 (Figure 4, Supplementary Figure S2) which was further supported by ACE value of 1884.7. After hindgut, the chao1 and ACE indices revealed higher species richness in the midgut (1,620 and 1,710) while least in the foregut (1,454 and 1,469). The phylogenetic diversity (PD) analysis of the hindgut metagenome having PD-tree value of 128.2, was significantly different from foregut metagenome (98) showing statistical significance of *p* < 0.005. Similarly, the Tukey HSD test based on Sob index revealed significant variation in alpha diversity (*p* < 0.01) of the bacteria in the gut-regions of *C. formosanus*. Significant differences in bacterial species richness and diversity were observed among the three gut-regions of the termite, in terms of an evaluation by Kruskal-Wallis test (*p* < 0.05).

**Figure 5:**
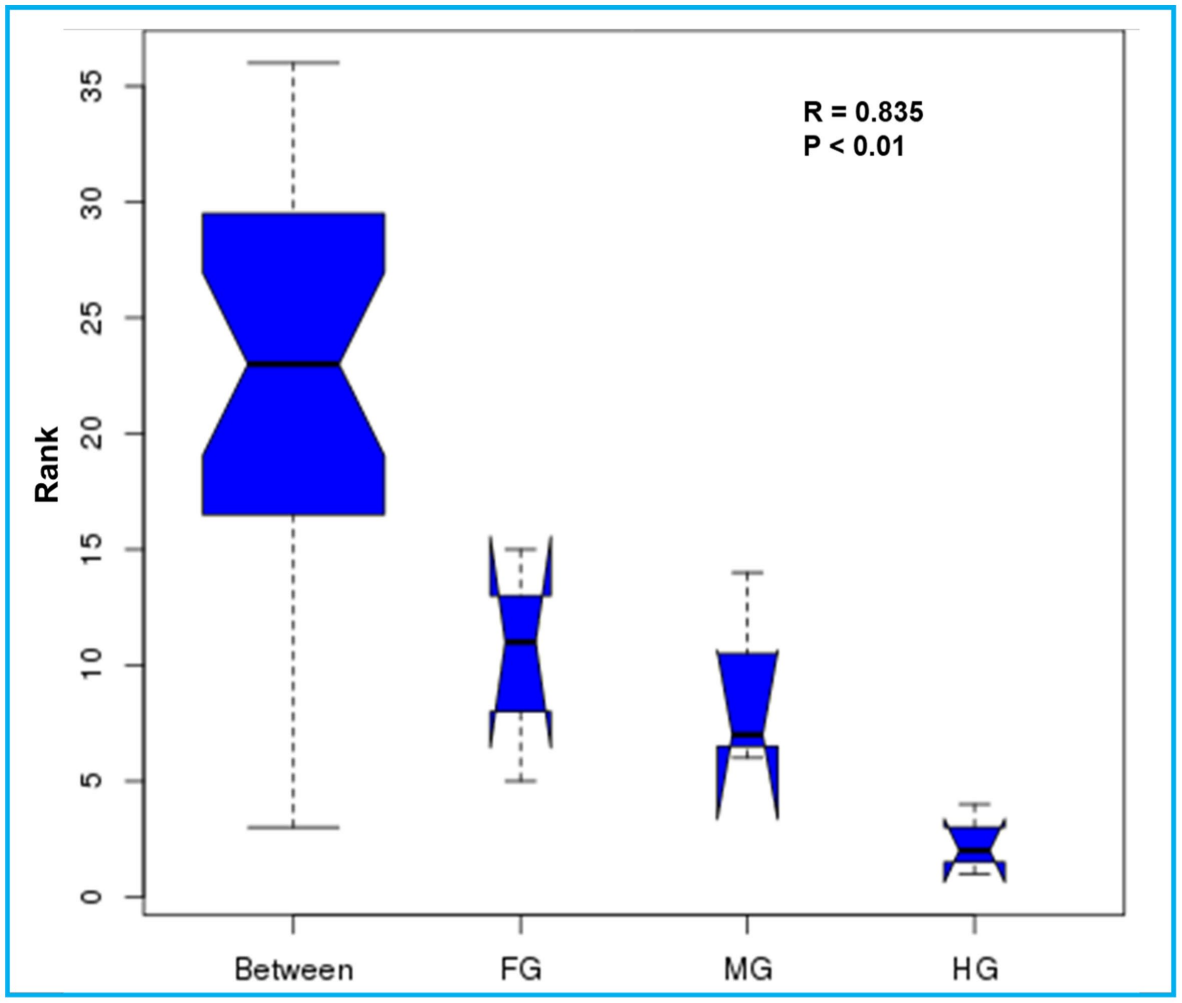
Similarity between the foregut, midgut and hindgut bacterial communities harbored by *C. formosanus.* Analysis of similarity (ANOSIM) box plot comparing the phylogenetic distances of the bacterial communities between the gut-regions of *C. formosanus* based on unweighted UniFrac distance.

**Figure 6:**
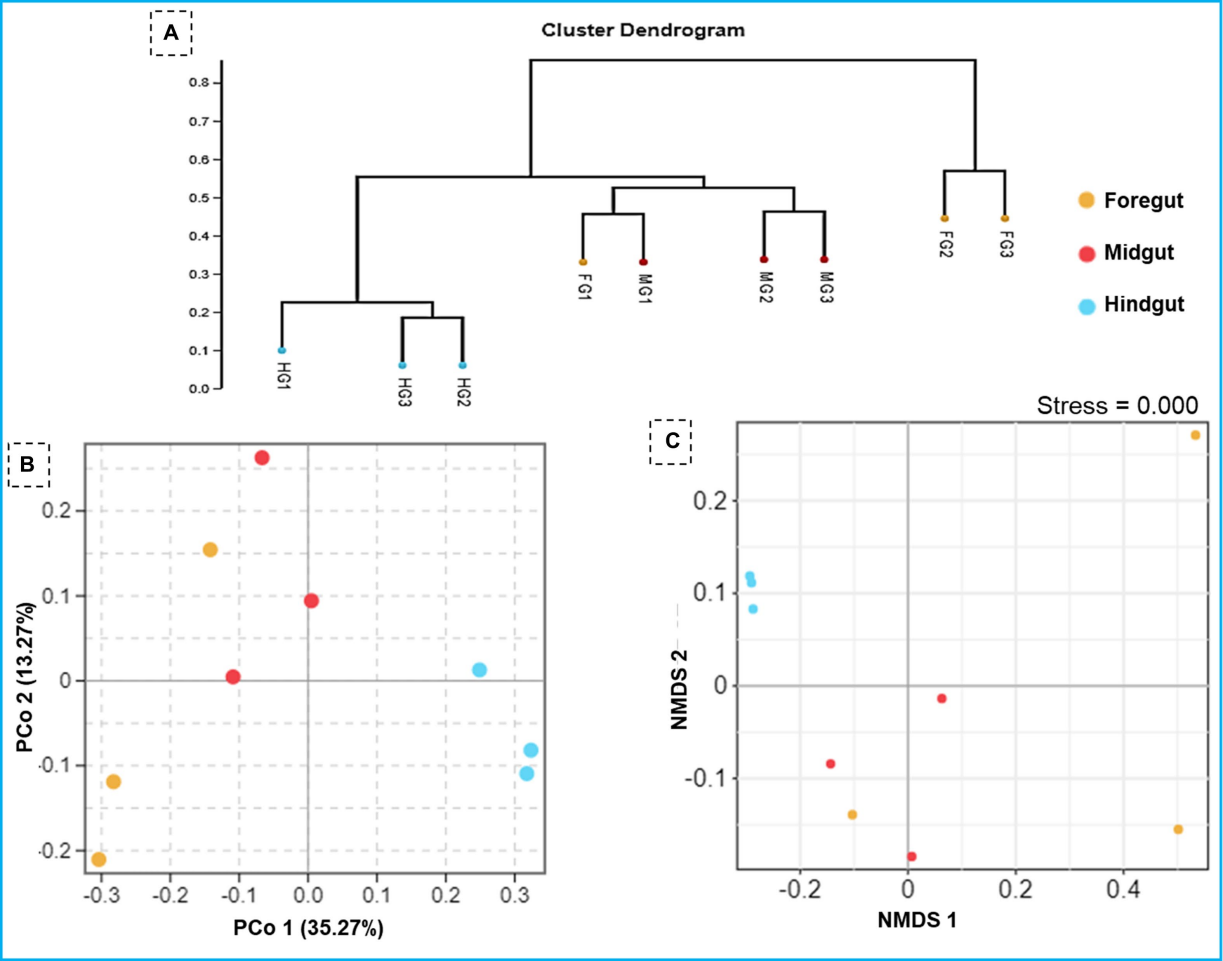
Similarity in the composition of bacterial community in three gut-regions of the termite, *C. formosanus*. **(A)** Representation of the UPGMA-based clustering dendrogram showing the distance matrix information between the samples while **(B,C)** depict principal coordinate analysis and nonmetric multidimensional scaling analyses of the bacterial communities in the gut system of the termite.

The ANOSIM R was equal to 0.835 (*p* < 0.01) which suggested differences in the intergroup similarity ratio for unweighted Unifrac distance (Figure 5, Table 3). The unweighted Unifrac distance-based NMDs analysis revealed higher intergroup distances than the intragroup variants (Figure 6). A similar trend (R2 = 0.4274, *p* < 0.01) was also shown by the unweighted-Unifrac distance based on the Adonis (PERMANOVA) analysis (Table 3). The weighted-Unifrac distance measurements (*R* = 0.547, *p* < 0.05) further indicated noticeable differences between intergroup and intragroup gut microbiomes. The PERMANOVA analysis (*p* < 0.01) based on the Unifrac distance matrices for bacterial community profiles confirmed that each of the gut-regions possessed unique bacterial diversity which might be due to the physiology and pH milieu of the gut environments.

**Table 3:** Differences between intragroup and intergroup unweighted Unifrac distances based on the Adonis (PERMANOVA) analysis.

Intergroup	DF	Sum of squares	Mean squares	*F* value	*R* ^2^	*p*-value
FG-*vs*-MG	1	0.170	0.170	1.23	0.23	0.2
FG-*vs*-HG	1	0.421	0.421	3.39	0.45	0.1
MG-*vs*-HG	1	0.267	0.267	2.18	0.35	0.1
FG-*vs*-MG-*vs*-HG	2	0.572	0.286	2.23	0.42	0.007*

DF, degree of freedom.

*Indicates statistically significant at *p* < 0.01.

### 3.3 Taxonomic structure of the bacterial community in *Coptotermes formosanus*

**Figure 7:**
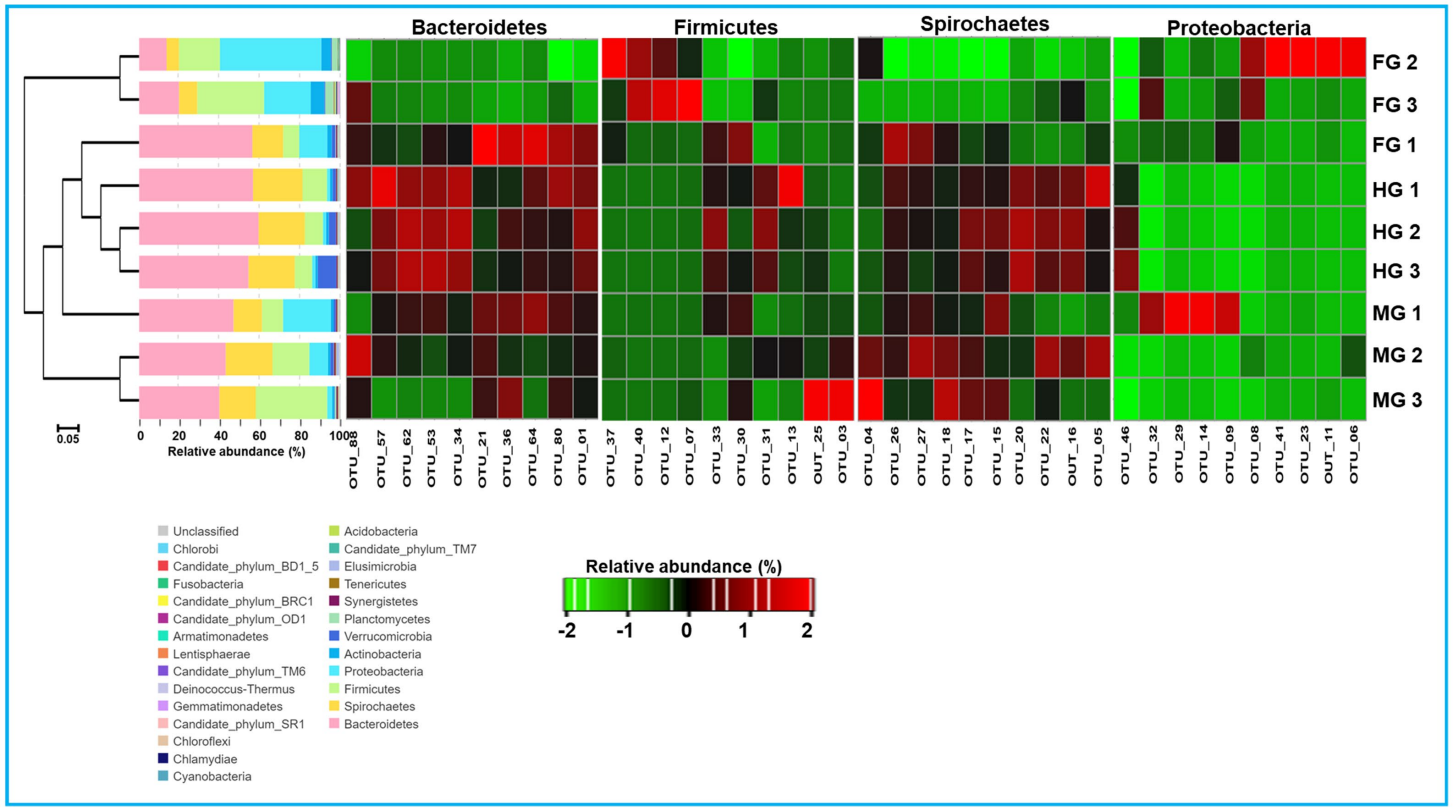
Taxonomic profiles of gut bacterial communities in the gut system of *C. formosanus*. A Cluster dendrogram calculated from the Bray–Curtis dissimilarity in bacterial community structure based on 16S rRNA gene amplicon sequencing, together with the distribution of OTUs into bacterial phyla and heat map representation of relative abundance of dominant OTUs from 4 major bacterial phyla in gut-regions of *C. formosanus*. ANOSIM R was equal to 0.877 with *p* < 0.05.

To characterize the phylogenetic affiliations of the bacterial communities associated with different gut-regions of the termite, *C. formosanus*, we analyzed gene sequencing of the community DNA. Overall, the identified bacteria were represented by 26 different phyla in the gut system of *C. formosanus*. All of the gut regions represented dominance of the Bacteroidetes with 30.2, 43.5, and 56.9%, respectively, in foregut, midgut and hindgut of the termite. However, after Bacteroidetes, the gut systems exhibited variable dominance of different phyla such as foregut, sheltered 28.9% of the Proteobacteria accounting for second dominant phylum in that region (Figure 7). In contrast, midgut and hindgut were dominated by Firmicutes and Spirochaetes with 21.2 and 23.3% abundance, respectively. In the foregut and midgut regions, the bacteria from 7 different phyla *viz.*, Bacteroidetes, Spirochaetes, Cyanobacteria, Firmicutes, Proteobacteria, Planctomycetes and Actinobacteria comprised over 97.73% and 96.58% of the total prokaryotic communities. However, the hindgut region was dominated by the members of Bacteroidetes, Spirochaetes and Firmicutes together with Verrucomicrobia that accounted for over 94% of the observed bacteria. One astonishing feature was the predominance of Verrucomicrobia in the hindgut where it contributed 4.78%, however its load in the foregut and midgut regions was very low (0.89 and 0.95% respectively). The members of the phylum Planctomycetes showed higher abundance in the foregut among the three gut-regions where it comprised over 2.39% as compared to midgut (0.33%) and hindgut (0.03%). Among the gut regions, the highest number of bacterial phyla were represented in midgut, i.e., 23 phyla followed by foregut that harbored the members of 22 bacterial phylotypes (Figure 7). However, bacteria belonging to only 18 phyla were represented in the hindgut signifying the autochthonous characteristic of some particular phylotypes like Bacteroidetes and Spirochaetes. The other phyla such as Actinobacteria, Verrucomicrobia, Synergistetes, Elusimicrobia and Tenericutes etc. were represented in lower fraction ranging from 0.03 to 4.8% in different regions of the gut of *C. formosanus*.

A total of bacteria affiliated to 142 families were observed in the gut system of *C. formosanus*, being highest, i.e., 120 families in the foregut, and then midgut (117 families). Out of the total observed families of bacteria, highest dominance was exhibited by Porphyromonadaceae being 53.5, 40.6 and 28.1% in the hindgut, midgut and foregut, respectively. After Porphyromonadaceae, the second most abundant family was Spirochaetaceae showing, 21.6, 16.5, and 9.0% in hindgut, midgut, and foregut, respectively. Similarly, the abundance of the Lachnospiraceae were higher in the midgut represented by 16.8%. The members of all other families such as Enterobacteriaceae, Moraxellaceae, Streptococcaceae, Caulobacteraceae, Rikenellaceae, etc. were found in lower numbers.

### 3.4 Analysis of the unique and enriched taxa specific to gut-regions

*Coptotermes formosanus* harbored a considerable diversity of bacteria belonging to 26 different phyla and 352 known genera. The members of the phyla like *Deinococcus thermus*, *Chlamydiae*, *Candidate phylum_TM6*, and *Armatimonadetes*, etc. were specific to particular gut regions (Supplementary additional file 1). The bacteria belonging to lineages of *Candidate_phylum*_TM6, and *Chlamydiae* were uniquely observed in foregut while the members of the bacterial groups such as *Deinococcus thermus,* and *Armatimonadetes* were shared by foregut and midgut regions.

**Figure 8:**
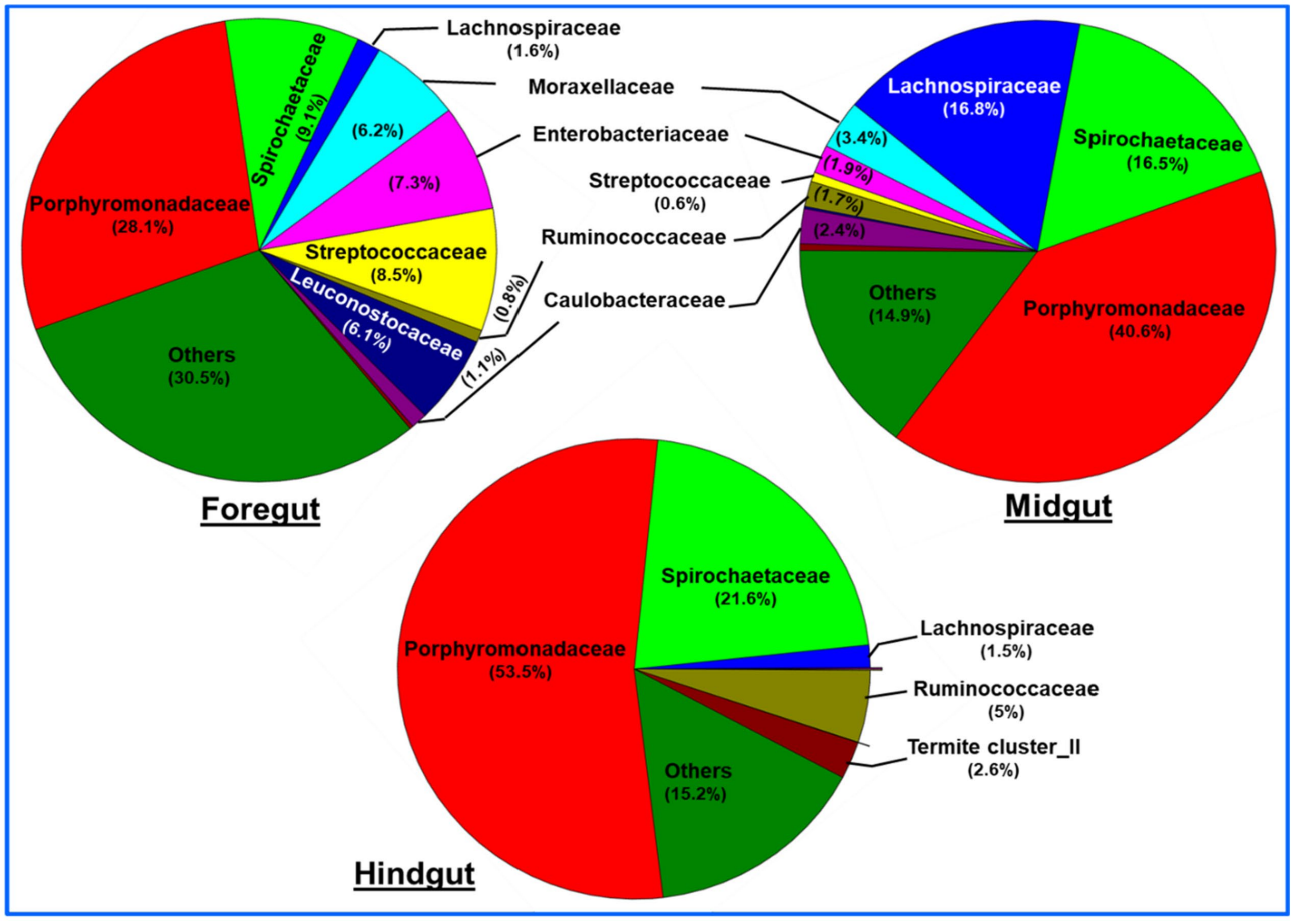
Region-wise distribution and abundance of the top 10 bacterial families in the gut system of wood-feeding termite, *C. formosanus*.

Similar to the phyla distribution, the lowest number (80) of bacterial families were observed in the hindgut. At the family level description, the top 10 families including Porphyromonadaceae contributed about 69.3% of the foregut bacteria (Figure 8). The midgut was dominated by Lachnospiraceae (16.8%) and Spirochaetaceae (16.5%) after Porphyromonadaceae (40.6%). Unlike foregut and midgut, the hindgut possessed members of 80 bacterial families being dominated by the Porphyromonadaceae (53.5%) and Spirochaetaceae (21.6%). The most dominant 10 families occupied about 84.9 and 84.7% of the bacterial families in the midgut and hindgut, respectively. The members of the families like Streptococcaceae, Enterobacteriaceae, and Leuconostocaceae were found more in the foregut region while species of Lachnospiraceae were uniquely abundant in the midgut region.

**Figure 9:**
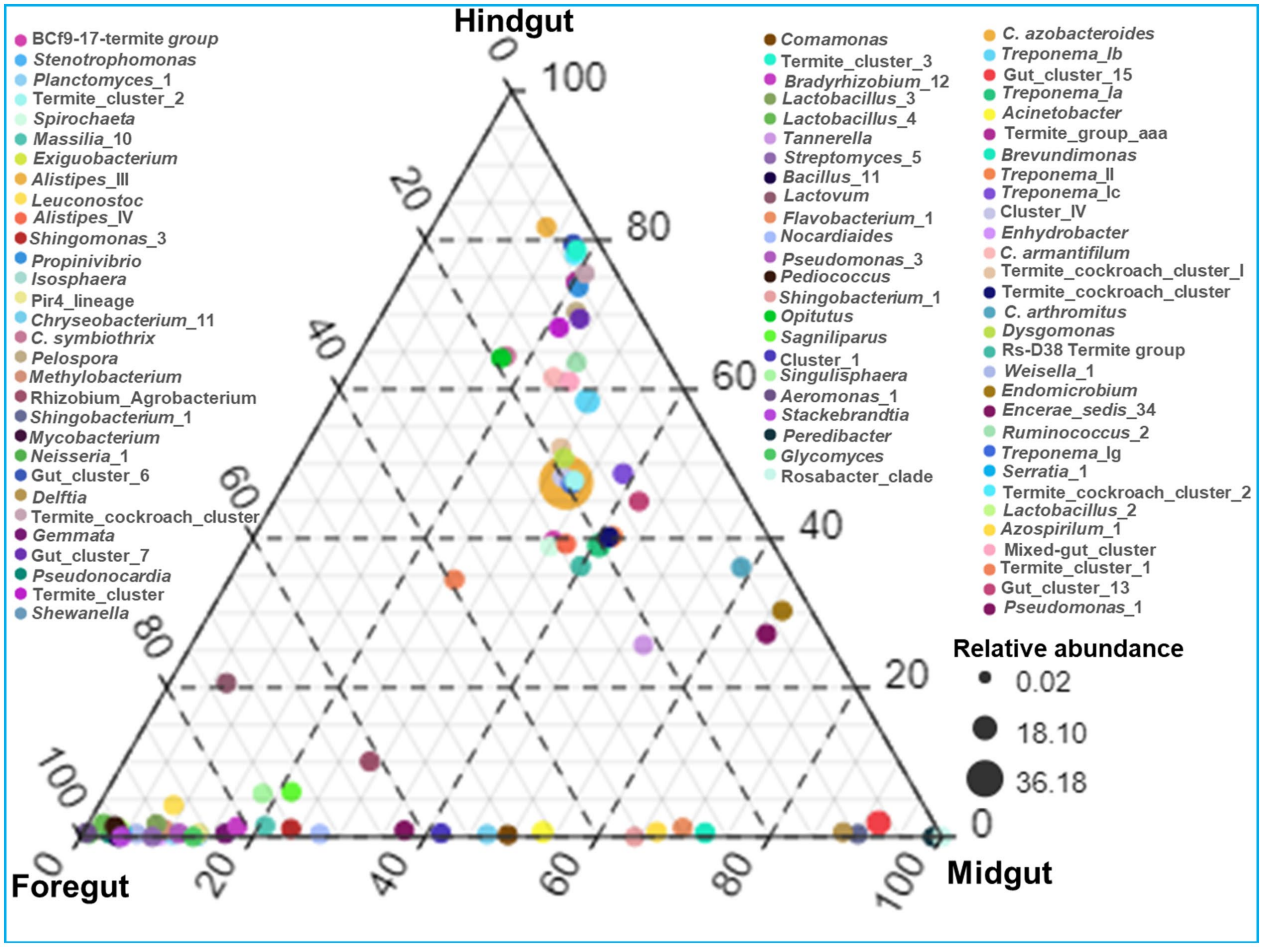
Genera-wise distribution and relative abundance of the bacteria in the gut-regions of the termite.

The identified bacteria from all the gut regions were found affiliated with 352 known genera apart from some unclassified species (Figure 9). The top 20 genera and their abundances in the gut-regions are shown in Table 4. At the genus level, *Candidatus* Azobacteroides was the most dominant throughout the gut system represented by 27.5, 39.8, and 51.7%, respectively, in the foregut, midgut, and hindgut. The Lactobacillaceae represented by *Weissella*, and *Lactobacillus* were majorly found in the foregut. Noteworthy, gut cluster 15 was highly abundant (15.8%) in the midgut after *Candidatus* Azobacteroides. However, the Moraxellaceae represented by *Acinetobacter* showed slight variations in the abundance between midgut (3.2%) and foregut (3.3%). The members of the genera like *Candidatus* and *Treponema* were most dominant in the hindgut showing 1.8 and 1.2-fold abundances when compared with foregut and midgut, respectively (Figure 9).

**Table 4:** Relative abundance of the top 20 bacterial genera in different gut-regions of the wood-feeding termite, *C. formosanus*.

Bacterial genera	Relative abundance %			Average relative abundance in whole gut (%)
	FG	MG	HG	
*Candidatus_azobacteroides*	27.5	39.8	51. 7	33.7
*Treponema*	6.9	12.7	15.9	9.8
Gut_cluster	1.8	15.8	1.9	8.8
*Acinetobacter*	3.3	3.2	<0.1	3.3
*Termite_group*	0.2	0.7	2.4	0.5
*Brevundimonas*	1.0	2.4	<0.1	1.7
*Enhydrobacter*	2.9	0.2	<0.1	1.5
*Candidatus_armantifilum*	0.4	0.5	1.3	0.5
*Candidatus_arthromitus*	<0.1	0.9	0.5	0.5
*Weissella*	1.0	<0.1	<0.1	0.5
*Dysgonomonas*	0.2	0.3	0.5	0.3
*Serratia*	0.9	<0.1	<0.1	0.5
*Rs-D38_Termite group*	0.2	0.4	0.3	0.3
*Lactobacillus*	0.8	<0.1	<0.1	0.4
*Incertae_Sedis_34*	<0.1	0.5	0.2	0.3
*Endomicrobium*	<0.1	0.5	0.2	0.3
*Ruminococcus*	0.1	0.2	0.4	0.2
*Stenotrophomonas*	0.6	<0.1	<0.1	0.4
*Planctomyces*	0.7	<0.1	<0.1	0.4
*Azospirillum*	0.2	0.5	<0.1	0.3
*Pseudomonas*	0.4	0.2	<0.1	0.3
*Alistipes*	<0.1	0.2	0.4	0.2
*Termite_cockroach_cluster*	0.3	0.8	0.9	0.5
Uncultured bacteria	1.6	1.6	2.4	1.6
Other genera	16.8	8.4	6.7	12.6

FG, foregut; MG, midgut; HG, hindgut.

**Figure 10:**
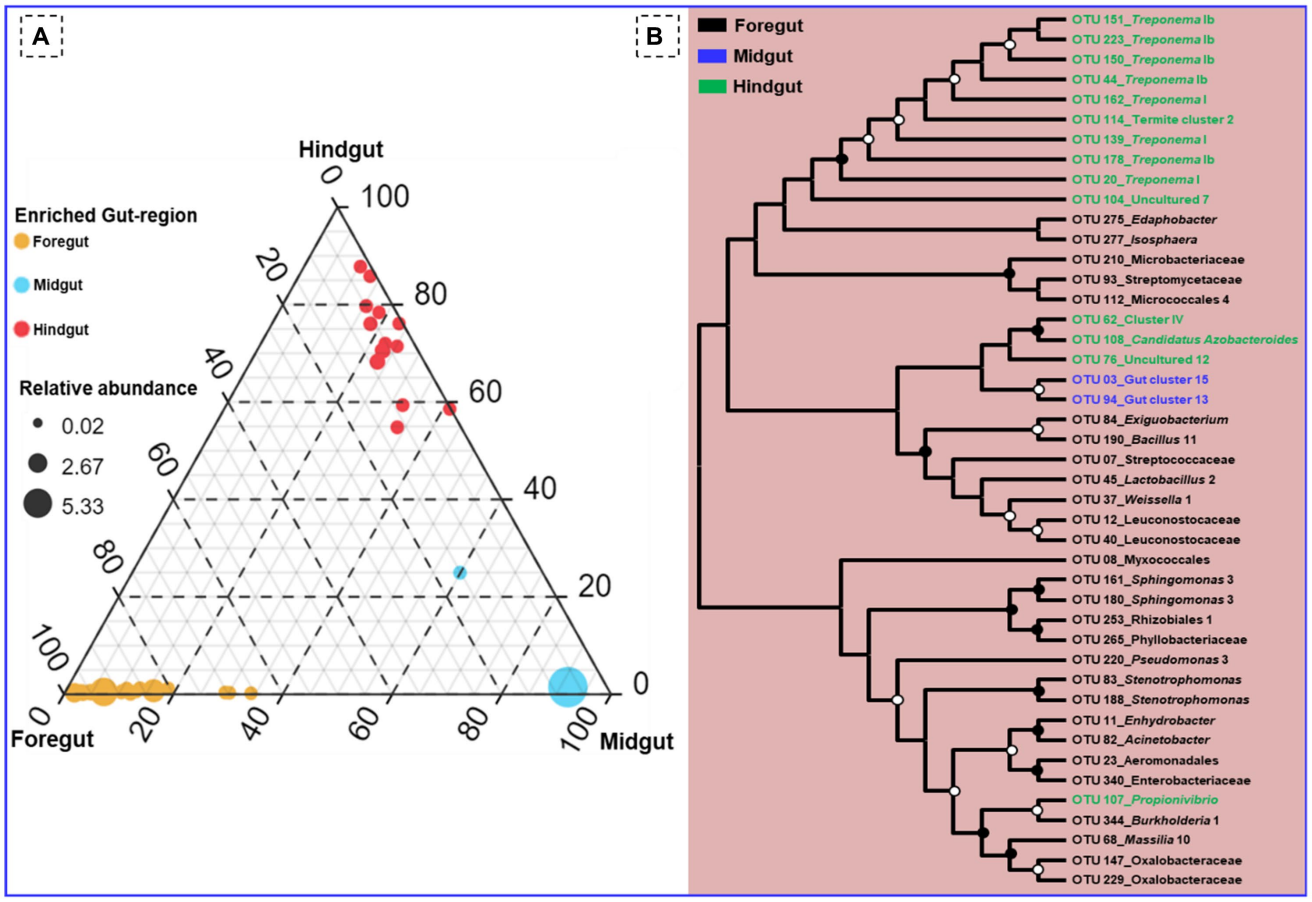
**(A)** Ternary plot demonstrating the enriched OTU particular to the gut region of the termite. **(B)** Phylogenetic tree revealing the taxonomic classification and relatedness of the enriched OTUs drawn on the basis of metagenomic sequences of the bacterial community in the gut-regions of the *C. formosanus*. Only nodes with bootstrap values based on 1,000 replicates >90% (●) and >50% (ο) are marked.

Among the gut-regions, Ruminicoccaceae was more abundant in hindgut that were classified as Termite group_aaa (OTU_13, OTU_73, OTU_79), Termite cockroach cluster (OTU_134), Gut_cluster_6 (OTU_152), Gut_cluster_7 (OTU_224), and uncultured_12 (OTU_76, OTU_33), *Ruminicoccus*_2 (OTU_71, OTU_116) (Supplementary additional file 2). The NCBI BLAST analysis for many OTUs from hindgut matched with uncultured *Treponema* 16S rRNA gene clones isolated from the gut of other termites such as *Reticulitermes speratus* and Formosan subterranean termite, etc. However, some *Treponema* represented OTUs showed confidence levels <90%, signifying the presence of possible novel *Treponema* phylotypes in *C. formosanus* (Figure 10).

Out of the total OTUs, only 44 OTUs were enriched in individual gut regions. Among these unique OTUs, majority 28 (63.6%) were sheltered in the foregut, while only 2 OTUs (OTU_03, and OTU_94) representing gut cluster_15, and gut cluster_13 from Lachnospiraceae were predominantly enriched in the midgut. The number of the unique OTUs enriched in the hindgut was 14 that were affiliated with Ruminicoccaceae, Termite gut cluster, Rhodocyclaceae, Porphyromonadaceae, Spirochaetaceae, Veillonellaceae besides some uncultured taxa (Figure 10). Among the 26 bacterial families that enriched in the gut system, the LEfSe indicated the enrichment of the Spirochaetaceae, Veillonellaceae, and Rhodocyclaceae in the hindgut while none of the families were enriched in the midgut (Supplementary Figure S3). The highest and most diverse bacteria were enriched in the foregut (23 families) probably due to its immediate contact with the surrounding environment ingested some environmental bacteria with food.

### 3.5 Putative functional profile of the bacteria toward lignocellulose digestion

**Figure 11:**
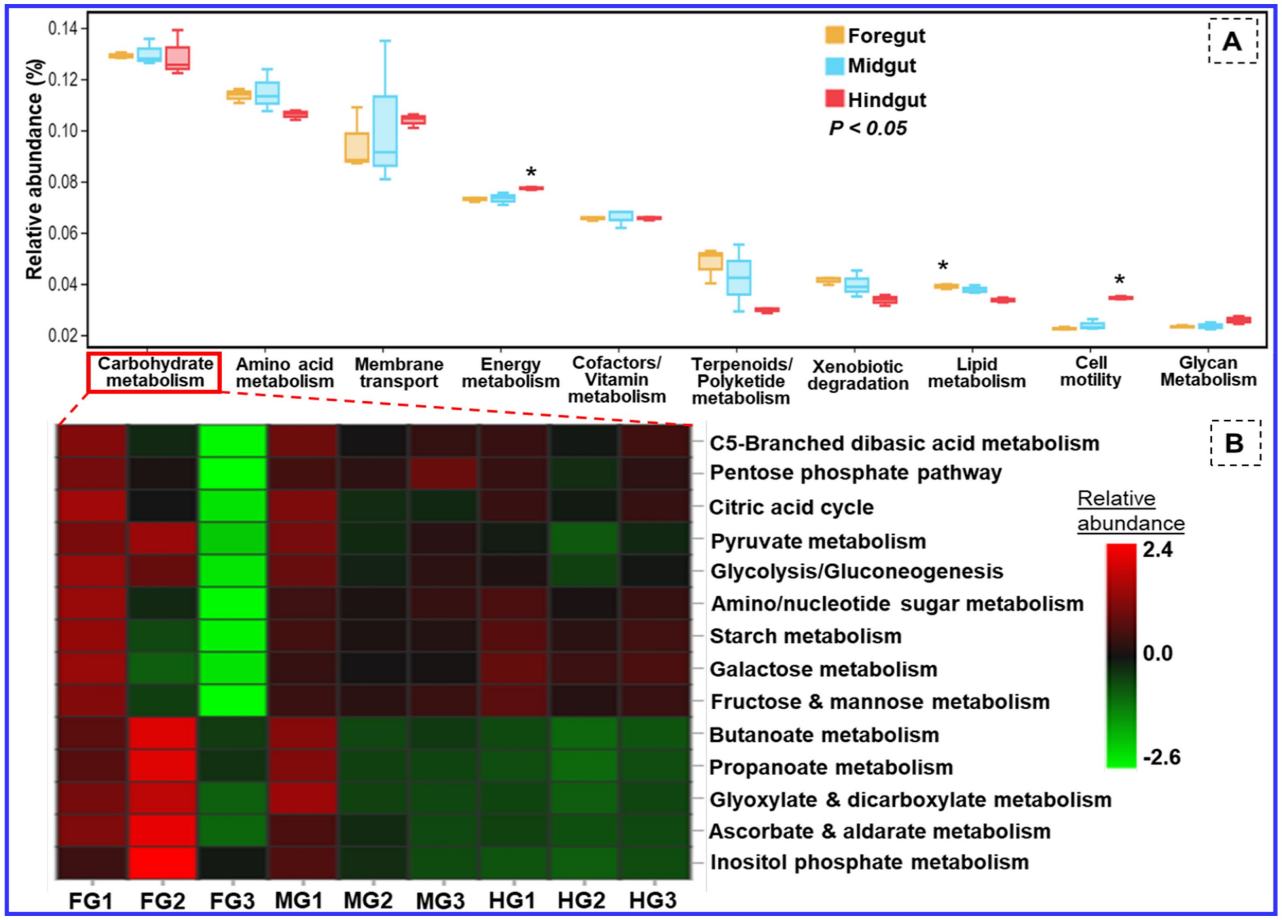
Putative functional profile of the bacterial symbionts in the gut of *C. formosanus*. **(A)** Metabolic pathway information of the bacterial symbionts based on KEGG prediction and KO abundance. **(B)** Heatmap of the relative abundance of modules related to carbohydrate metabolism identified in prokaryotic microbiomes of *C. formosanus*. *p ≤ 0.05 indicates statistical significance of particular functions of bacteria between gut-regions based on Tukey HSD test. The abbreviations FG, MG, and HG denote the 3 sample replicates each of the foregut, midgut, and hindgut regions of the termite under consideration.

The functional profiling of the metagenomic data revealed shared metabolic signatures of bacterial communities residing in the foregut, midgut, and hindgut of *C. formosanus*. The bacteria showed a diverse array of symbiotic functions, such as carbohydrate and amino acid metabolisms, detoxification, and degradation of xenobiotics and terpenoids besides many other pivotal roles (Figure 11). The carbohydrate and energy metabolisms were perpetual processes for the gut microbiota system of the lower wood-feeding termite, showing 12–13% and ~7% relative abundances among the gut-regions, respectively (Figure 11). For energy metabolism, the Tukey’s HSD test was significant when the functions of bacteria from hindgut were compared with foregut and midgut regions (*p* < 0.05). However, the relative abundance of different metabolic modules assigned to the carbohydrate metabolism (Figure 11B) was statistically insignificant among the gut-regions indicating it as a continuous process.

### 3.6 Symbiosis of the bacteria with the host for other metabolic functions

Besides energy and glycan metabolisms, the bacterial communities of the hindgut were chiefly involved in cellular processes like signal transduction and cell motility. Since the foregut acts as the main recipient of the environmental bacteria it showed higher (>4.1%) relative abundance toward detoxification mechanisms like xenobiotic and terpenoid degradation. The relative abundance of lipid metabolism was above 3.8 and 3.7% in the anterior gut (foregut and midgut) when compared to the hindgut which showed a lower amount of sequence reads, i.e., 3.3% (Figure 11). The functional profiles calculated from the KEGG Orthology (KO) database of prokaryotes revealed sequence reads that encode for environmental adaptation were predominant in the hindgut with a relative abundance of 0.0025. The BugBase analysis demonstrated a high prevalence of the gram-positive and biofilm-forming bacteria in the midgut (4.2%), followed by the foregut (3.9%) while the hindgut showed a higher abundance of aerobic, and gram-negative bacteria affiliated with Bacteroidetes (Supplementary Figure S4). The foregut being a major hub for reception of the environmental microorganisms showed predominance of anaerobic/facultative anaerobes and mobile-element-containing bacteria having high stress tolerance potentially oxidative stress imposed by the lignin contents of the diet besides the toxicity caused by some pathogens ingested from the environment. The representation of the gram-positive bacteria was observed least in the hindgut. The major community of the biofilm-forming bacteria was comprised of the members belonging to the phyla of Spirochaetes, Proteobacteria, and Firmicutes from midgut (Supplementary Figure S3E). Likewise, most of the Bacteroidetes members formed anaerobic consortium that were found abundant in the hindgut, however, least in the foregut.

## 4 Discussion

The gut microbial symbionts are known to provide a diverse array of services to the host including eusociality, nutrition, immunity, and digestive symbiosis (Warnecke et al., 2007; Dar et al., 2021a,b, 2022, 2024; Zhang et al., 2023). During the last three decades, a plethora of information has been accumulated regarding the diversity and functions of microbiota within termite gut systems (Sun et al., 2014; Brune and Dietrich, 2015; Scharf and Peterson, 2021). Despite numerous studies characterizing hindgut microbial communities, there remains a notable gap in our understanding of the structure and complexity of bacteria in individual gut-regions, particularly the foregut, and midgut of termites. Moreover, the functions of symbiotic bacteria in specific gut-regions of termites are still not well-defined. To this end, we demonstrate the variety of microbial communities sheltered in individual gut-regions of the wood-feeding termite, *C. formosanus* by dissecting over 1,000 gut samples from worker termites and analyzed their metagenomes to identify bacterial symbionts. The observed results demonstrate significant differences in the composition of bacterial communities across the foregut, midgut, and hindgut regions of the wood-feeding termites. This report contributes to the existing knowledge of gut bacterial communities, particularly in previously understudied foregut and midgut regions, besides shedding light on the metagenomics of the hindgut in wood-feeding lower termites. Importantly, this study represents the first comprehensive investigation into the diversity and composition of bacterial symbionts across the spatial structure of the gut system of wood-feeding lower termites, particularly *C. formosanus*. In this perspective, the present study may serve as the basis for future research focused on the localization, characterization, diversity, and functional analysis of termite gut bacterial symbionts.

The generated metagenomic libraries revealed that *C. formosanus* harbors a diverse array of bacterial symbionts in its gut system. The cumulative abundance of Bacteroidetes, Spirochaetes, Firmicutes, Proteobacteria, and Actinobacteria in all the three gut-regions of *C. formosanus* were 43.5, 17.2, 17.1, 14, and 2.3%, respectively. Notably, the abundance of Bacteroidetes in our data sets (30.2, 43.5, and 56.9% in the foregut, midgut, and hindgut respectively) exceeded that observed in some lower termites (Dietrich et al., 2014), although, it was comparatively lower than the prevalence in closely related species like *Coptotermes niger* (Noda et al., 2005) and other termites (Nakajima et al., 2006; Liu et al., 2013; Makonde et al., 2013). The abundance of other phyla that are metabolically diverse such as Elusimicrobia, and Synergistetes was almost similar among the termite’s gut-regions (Méheust et al., 2020). However, the absence of Fibrobacteres is surprising as it constitutes a prevalent member involved in rumen fermentations (Hongoh et al., 2006; Mikaelyan et al., 2015, 2017). The gut bacteria of *C. formosanus* can be categorized into 8 dominant families when considering their relative abundance of over 5% (Figure 8). At the family level, Porphyromonadaceae, Spirochaetaceae, Lachnospiraceae, Moraxellaceae, Enterobacteriaceae, Ruminicoccaceae, and Streptococcaceae exhibited the highest species richness across all three gut-regions. The majority of the taxa of these bacterial families prefer gut systems of termites and invertebrates, comprising a diverse array of genus-level lineages (Bourguignon et al., 2018; Huang et al., 2022; Zhang et al., 2024). The dominance of Porphyromonadaceae is consistent with other lower termites such as *Stolotermes ruficeps* (Reid et al., 2014). Most of the bacterial lineages, particularly Porphyromonadaceae, Ruminococcaceae, and Spirochaetaceae are typically associated with hindgut compartments, and high pH in cockroach gut that share a common ancestor with termites (Lampert et al., 2019). Unlike the dominance of *Treponema* cluster Ic and If that are exclusive to higher termites (Köhler et al., 2012; Dietrich et al., 2014; Rahman et al., 2015; Tokuda et al., 2018), the higher abundance of *Treponema*_Ib and _Ia in *C. formosanus* corroborates with the consistency of these bacterial lineages in wood-feeding termite species like *Globitermes brachycerastes* (Liu et al., 2019). Interestingly, few OTUs (OTU_17, OTU_58, and OTU_127) representing *Treponema* cluster Ic and *Treponema*_Ig lineages were found in the hindgut of the termite which is surprising and necessitates further research to comprehensively evaluate their co-speciation in termites. Many OTUs classified as Rs-D38 termite group affiliated with Rikenellaceae were prevalent throughout the gut system, exhibiting higher expression in the hindgut. The occurrence of Rs-D38 group in cockroaches and termites, especially in *S. ruficeps* (Stolotermitidae) and *Incisitermes marginipennis* (Kalotermitidae), emphasizes its adaptability to diverse habitats (Ware et al., 2010; Dietrich et al., 2014). Most of the Lachnospiraceae sequences affiliated with *Incertae sedis* 34 (OUTs_42), *Candidatus* Arthromitus (OTU_25, OTU_171), and Termite gut clusters (OTU_03, OTU_94, OTU_117) displayed higher abundance in the midgut. These lineages are generally less abundant in cockroaches, and many lower termites but are predominant in some higher termites (Kohler, 2011).

The Synergistaceae, represented by the Termite cockroach cluster, was not among the most abundant families, however, it demonstrated a uniform distribution across the gut systems. Many studies have suggested that this family is underrepresented in DNA data sets (Reid et al., 2014), while others highlighted its presence with *Candidatus* Tammella, playing crucial roles in amino acid fermentation within termite gut systems (Nakajima et al., 2006; Hongoh et al., 2007b). Though low in abundance, Tenericutes also exhibited uniform distribution among the gut-regions. This aligns with recent findings indicating the prevalence of Tenericutes in various termite and cockroach species (Dietrich et al., 2014; Reid et al., 2014). Nevertheless, the fate and distribution of major lineages like *Candidatus* Azobacteroides, *Candidatus* Armantifilum, *Treponema*_Ia, and *Treponema*_Ib, etc. that are present in all the three gut-regions (though with variable abundance) is worth further investigation and thorough attention. Irrespective of the absence of flagellates in the anterior gut-regions, these lineages persist throughout the entire gut system, suggesting that physiology, processed diet, and microenvironmental conditions play pivotal roles in the distribution and selection of these bacteria in termites (Köhler et al., 2012; Mikaelyan et al., 2015, 2017). Moreover, our analysis suggests that these bacteria might differ considerably with regards to species or strain level bacterial lineages in the case of the termites feeding on the same diet and across the gut system of the same hosts. These differences may further reflect their unique functions toward the metabolism of lignocellulose or other physiological processes of the host.

### 4.1 *Coptotermes formosanus* harbors gut-region specific bacterial taxa

Indeed, our data showed that the composition of symbiotic bacteria in *C. formosanus* varies significantly concerning the structure as well as gut physiology of the termite. The highest α-diversity was observed in the foregut followed by the hindgut as revealed by Shannon and Simpson indices. This could be attributed primarily to the foregut’s exposure to the environmental bacteria ingested through food (Bignell et al., 2011). The higher diversity of bacterial symbionts in the hindgut is due to its large size and available surface area provided by flagellates, accommodating endo and ecto-symbionts (Smith et al., 2017; Nalepa, 2020). Flagellates also influence the composition of bacterial communities in termite guts (Reid et al., 2014). Several species of Verrucomicrobia*, Candidate phylum*_SR1, *Candidate phylum*_BD1-5 besides Bacteroidetes, and Spirochaetes, dominant in the data sets of hindguts are well-known as ecto- and endo- symbionts of flagellates in termites (Hongoh et al., 2007a, 2008a; Sato et al., 2014, 2009). Notably, bacterial lineages like Porphyromonadaceae cluster_V, Spirochaetaceae_*Treponema* I, and Pseudomonadaceae, identified as endosymbionts of *C. formosanus* residing within the cells of cellulolytic protist *Pseudotrichonympha grassii*, have been associated with high redox potential in other termites and Surinam cockroach, *Pycnoscelus surinamensis* (Hongoh et al., 2008a; Lampert et al., 2019). Additionally, many sequences (OTU_135) from the hindgut represented by bristle-like uncultured bacteria were classified as *Candidatus* Symbiothrix, a taxon exclusive to termite guts, acting as ectosymbionts of protists coexisting with Spirochaetes (Hongoh et al., 2007a). Although we observed a low abundance of *Candidatus* Symbiothrix in *C. formosanus,* it has been reported as highly dominant in other termites, including *C. niger* (Kohler, 2011; Reid et al., 2014). The observed low abundance of OTUs representing *Candidatus* Symbiothrix could be attributed to different geographical distributions and evolutionary history, as *C. niger* is native to neotropics having distinct biogeographical attributes (Chouvenc et al., 2016).

The spatial structure of the bacteria in *C. formosanus* also showed an abundance of Proteobacteria (28.9%), Actinobacteria (4.5%), and Planctomycetes (2.3%) in the foregut. This could be attributed to their procurement from the environment through wood particles as Proteobacteria is the most diverse and largest bacterial group in the environment (Guimaraes et al., 2020). The predominance of these bacterial phylotypes aligns with previous findings (Utami et al., 2018; Dar et al., 2022), suggesting their symbiosis and functional roles in *C. formosanus*. These inferences are further evidenced by our previous observations that demonstrated Proteobacteria, as the dominant culturable phylotype in the termite’s gut-regions (Dar et al., 2022). Among the Proteobacteria, members of the Enterobacteriaceae and Moraxellaceae of the class Gamma proteobacteria were well represented across the foregut (7.39 and 6.26%) and midgut (1.9 and 3.46% relative abundance). Similarly, Lachnospiraceae (16.8%), and Caulobacteraceae (2.43%) were more abundant in the midgut as compared to the hindgut among the dominant families of class Clostridia, and Alphaproteobacteria, respectively. The abundances of Enterobacteriaceae and Moraxellaceae have been influenced by factors like low pH and high partial pressure of hydrogen ions in the cockroach gut (Lampert et al., 2019). Majority of the OTUs representing Actinobacteria (OTUs_93, OTU_185, OTU_48, OTU_144, and OTU_195) from *Streptomyces* lineage, displayed higher abundance in the foregut, reflecting their role in secreting antibiotics and secondary antimicrobial compounds to inhibit incoming pathogens in termite guts (Visser et al., 2012; Arango et al., 2016). The foregut of *C. formosanus* was also densely colonized by several deep-branching lineages of Planctomycetes (such as *Planctomyces*_1*, Isosphaera, Gemmata*), akin to the hindgut of soil feeding higher termite, *Cubitermes* spp. (Köhler et al., 2008).

The physicochemical microenvironment of the gut has been recognized as a strong selective factor that determines the composition of the gut-region-specific bacterial lineages in termites (Bauer et al., 2015; Mikaelyan et al., 2017). Alterations in intestinal pH induce modifications in microbial composition, reducing or eliminating the pathogenic bacteria sensitive to acidic conditions while favoring acid-resistant microbes (Mroz et al., 2002). The higher presence of the *Lactobacillus* (OTU_45, OTU_175, and OTU_176) in the foregut corroborates with their role in the putative fermentation of ingested sugars created due to maceration of wood leading to the slightly acidic environment. However, the low amount of *Lactobacillus* in the midgut and hindgut could be attributed to low concentrations of lactate caused by the high turnover of the compound (Tholen and Brune, 2000). Moreover, the microbial activities in gut compartments give rise to steep radial gradients of oxygen, hydrogen, and potentially other metabolites that in turn determine the community structure of symbiotic bacteria (Kappler and Brune, 2002; Köhler et al., 2012; Ngugi and Brune, 2012). It has been hypothesized that the [FeFe] hydrogenases and putative [FeFe] hydrogen sensors of *Treponema primitia*, whose homologs are abundantly represented in the metagenomes of *Nasutitermes corniger* (Warnecke et al., 2007), are not only involved in reductive acetogenesis but also assist spirochetes in locating optimal positions within hydrogen gradients (Ballor et al., 2012).

Another factor that can rapidly change the microbial profile in termite gut is diet (David et al., 2014; Mikaelyan et al., 2015). Progressive changes in the availability and complexity of polysaccharides in lignocellulose during gut passage (Tokuda et al., 2018), together with a longer retention time of digesta in the hindgut, contributes to the relative abundance of specialized taxa like Bacteroidetes (Mikaelyan et al., 2017). Furthermore, variations in the chemical composition of the digesta along the gut passage (e.g., predominant oxidation of lignin in the foregut and midgut) also influence the structure of the microbial communities (Coy et al., 2010). These observations strongly support the notion that niche heterogeneity within microbial environments is a key determinant of community structure (Yang et al., 2005) and aligns with the unique diversity observed in different gut fractions of *C. formosanus*.

### 4.2 Bacterial symbionts contribute to the digestion of *Coptotermes formosanus*

**Figure 12:**
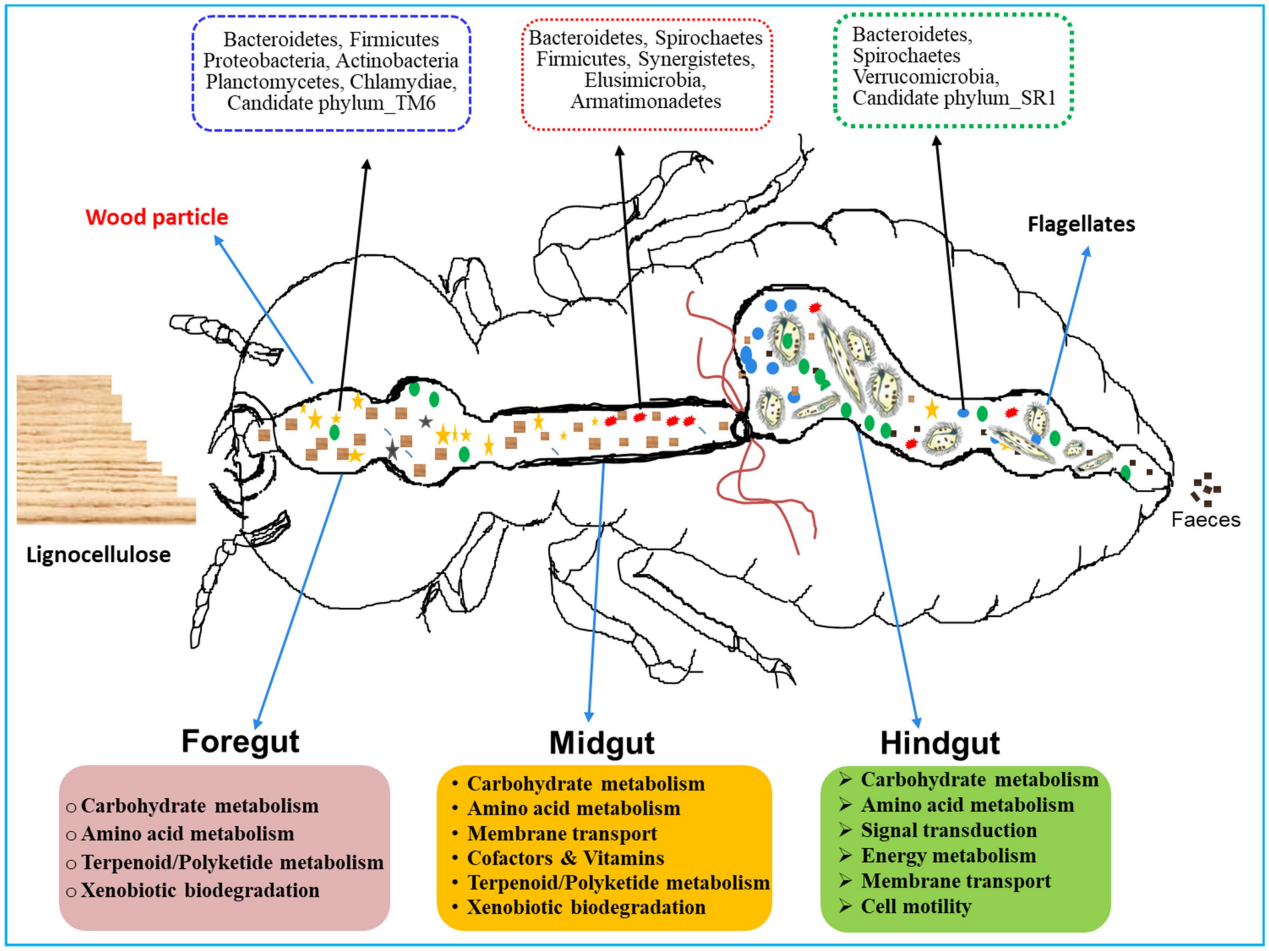
A proposed model for region-wise diversity and abundance of bacterial symbionts in the gut system of wood-feeding termite, *Coptotermes formosanus*, where they perform a variety of functions for the host in a collaborative fashion.

*Coptotermes formosanus* being a socio-economic pest of wood, efficiently degrades the lignocellulose-based diets in collaboration with gut symbionts including bacteria (Shinzato et al., 2005; Geng et al., 2018b; Dar et al., 2022). Previous studies have reported that Proteobacteria and Actinobacteria, significantly contribute to the lignocellulose digestion in the hindgut of *Holotrichia parallela* larvae (Huang et al., 2012). Consequently, the predominance of these bacteria in the foregut and hindgut of *C. formosanus* suggests their potential roles in plant cell wall digestion, thereby supplying energy and nutrients to the host (Huang et al., 2012). Similarly, the occurrence of Bacteroidetes throughout the gut system, with higher abundance in the hindgut of *C. formosanus* (Figure 12), aligns with their recognized involvement in fermentative metabolism and hydrolysis of plant-derived oligosaccharides (Hongoh et al., 2008b; Ghanbari et al., 2015). The higher abundance of Bacteroidetes in the hindgut also corresponds to their obligately anaerobic nature, underscoring their proficiency in polysaccharide fermentation, generating essential molecules such as acetate, and butyrate, absorbed by the host (Pan et al., 2023). Moreover, Spirochaetes are lauded for complementing reductive acetogenesis and nitrogen fixation activities in termites (Tokuda, 2021). The gram-positive, *Leuconostoc* is advantageous to the host by fermenting a variety of polysaccharides, mannitol, vitamins K, and bacteriocins, and catalyzes the hydrolysis of α-galactosides (Weymarn et al., 2002; Sybesma et al., 2003; Hemme and Foucaud-Scheunemann, 2004). Recently *Leuconostoc pseudomesenteroides* has been found to restore intestinal disorders induced by high-fat diets (Sun et al., 2020). The elevated abundance of these phylotypes in the anterior gut of *C. formosanus* signposts their involvement in lignocellulose digestion and fermentation, alongside potential roles in maintaining intestinal homeostasis. Further, the higher abundance of bacterial lineages like uncultured members of the “*Treponema* I” clade, Cluster_IV, *Dysgonomonas*, *Candidatus* Armantifilum, *Candidatus* Azobacteroides, *Ruminococcus*_2, and the Termite_cockroach_cluster, etc. that represent the lignocellulolytic community (Tokuda et al., 2018) throughout the gut system signifies the continuous degradation of the lignocellulose by the termite. Nonetheless, the low abundance or absence of some lineages among the gut-regions could be attributed particularly to the changes in the structure and composition of the processed lignocellulose-based diet besides the microenvironmental conditions and presence of flagellates in the termite gut system (Benjamino et al., 2018).

### 4.3 Bacterial symbionts augment the nitrogen metabolism and defense of the termites

The gut microbiota not only aids in the digestion of plant fiber but also plays a crucial function in the nitrogen economy and immunity of the termites. Hitherto, numerous nitrogen-fixing bacterial strains have been identified from termite guts, though a majority of them are unculturable. The enormous diversity of nifH genes observed in termite guts stipulates that nitrogen fixation capabilities are attributed to Spirochaetes, Clostridiales, Bacteroidetes, and other gut-bacterial symbionts (Lilburn et al., 2001; Warnecke et al., 2007; Yamada et al., 2007; Hongoh et al., 2008b; Desai and Brune, 2012), predominantly observed in the gut-metagenomes of *C. formosanus* (Figure 12). Our results are congruent with previous findings indicating that the endosymbiont, *Candidatus* Azobacteroides *pseudotrichonymphae* (Noda et al., 2007; Hongoh et al., 2008b) serves as the most significant diazotroph in the gut of *C. formosanus*, that possess nif genes. The presence of the diazotroph, *Candidatus* Azobacteroides, and Spirochetes like *Treponema* spp. throughout the gut system of *C. formosanus* also signposts its importance in nitrogen metabolism of the termite (Figure 9) as these symbionts are known to express paralogs of *nifH* genes (Desai and Brune, 2012; Tokuda, 2021). Recent identifications of many nitrogenase-encoding genes from Treponematales and Enterobacteriaceae in higher termite gut metagenomes further substantiate these findings (Arora et al., 2022). Moreover, termite guts contain nitrogenous residues derived from the cell biomass of bacteria (He et al., 2013; Liu et al., 2019), therefore, multiple KOs relevant to protein degradation, amino acid metabolism, cofactors, and vitamin metabolisms were enriched in the metagenomes of termite gut, particularly in the foregut and midgut. The anterior gut is mainly responsible for the elimination of environmental and allochthonous bacteria. Noteworthy, the dominance of Firmicutes in the anterior gut (foregut and midgut) of *C. formosanus* signifies its possible involvement in protein degradation, maintenance of gut homeostasis, and the development of host immunity (Colston and Jackson, 2016). Furthermore, the higher abundance of biofilm-forming bacteria in the anterior gut regions confers advantages to the termite by fortifying host defenses, augmenting nutrient exchange, and preventing the colonization of pathogens (Tytgat et al., 2019). The unique occurrence of the *Deinococcus* and *Armatimonadetes* in the anterior gut (foregut and midgut) could be augmenting the metabolism of terpenoids and polyketides and preventing stress from the secondary metabolites present in the wood (Gerber et al., 2015).

## 5 Conclusion

In this study, we have described the biodiversity of bacteria within the less studied gut regions of wood-feeding lower termite. Our findings suggest that despite feeding on a single diet (wood) there is a high degree of uniqueness among the phylogenetic lineages of bacteria within the gut systems of *C. formosanus*. The high specificity of the bacterial symbionts in different gut compartments of the termite provides strong evidence that microbial communities might be shaped by the environmental factors prevailing in the respective microhabitats. Further, the variable distribution pattern of the bacterial phylotypes among the gut-regions indicates unique functions toward the host physiology particularly in the complex processing of lignocellulose digestion and energy metabolism. Although the metagenomic analysis provided thorough insights into the bacterial diversity residing in the individual gut-regions of *C. formosanus*. A combined meta-analysis based on the metagenomic and metatranscriptomic research is inspired to better understand the interactions between host and microbiota, toward the functions in the gut system of *C. formosanus*. Moreover, for the present study, termites were collected from only one location and fed on a single diet. Feeding termites on different diets including antibiotic-treated wood together with collection from different geographical areas might help to decode the evolutionary pattern of symbiosis and enhance our understanding of the functionalities of these microbiomes within individual gut-regions of the termite. Nonetheless, the observed results are encouraging and provide the basis for a thorough analysis of the compartment-specific interplay existing between the microbiota and the host toward the complex process of lignocellulose digestion.

## Data availability statement

The datasets presented in this study can be found in online repositories. The names of the repository/repositories and accession number(s) can be found in the article/Supplementary material.

## Ethics statement

The manuscript presents research on animals that do not require ethical approval for their study.

## Author contributions

MD: Conceptualization, Investigation, Methodology, Writing – original draft. RX: Data curation, Formal analysis, Investigation, Methodology, Validation, Writing – original draft, Writing – review & editing. LJ: Data curation, Resources, Software, Visualization, Writing – review & editing. XQ: Formal analysis, Investigation, Resources, Software, Visualization, Writing – review & editing. SA: Formal analysis, Methodology, Resources, Software, Validation, Writing – review & editing. RP: Formal analysis, Resources, Supervision, Validation, Visualization, Writing – review & editing. CS: Data curation, Formal analysis, Methodology, Resources, Software, Validation, Visualization, Writing – review & editing. JS: Funding acquisition, Project administration, Supervision, Validation, Writing – review & editing.

## References

[ref1] Apprill A. McNally S. Parsons R. Weber L. (2015). Minor revision to V4 region SSU rRNA 806R gene primer greatly increases detection of SAR11 bacterioplankton. Aquat. Microbial. Ecol. 75, 129–137. doi: 10.3354/AME01753

[ref2] Arango R. A. Carlson C. M. Currie C. R. McDonald B. R. Book A. J. Green F.. (2016). Antimicrobial activity of actinobacteria isolated from the guts of subterranean termites. Environ. Entomol. 45, 1415–1423. doi: 10.1093/ee/nvw126, PMID: 28028088 PMC5225880

[ref3] Arora J. Kinjo Y. Šobotník J. Bucek A. Clitheroe C. Stiblik P.. (2022). The functional evolution of termite gut microbiota. Microbiome 10:78. doi: 10.1186/s40168-022-01258-3, PMID: 35624491 PMC9137090

[ref4] Aßhauer K. P. Wemheuer B. Daniel R. Meinicke P. (2015). Tax4Fun: predicting functional profiles from metagenomic 16S rRNA data. Bioinformatics 31, 2882–2884. doi: 10.1093/bioinformatics/btv287, PMID: 25957349 PMC4547618

[ref5] Ballor N. R. Paulsen I. Leadbetter J. R. (2012). Genomic analysis reveals multiple [FeFe] hydrogenases and hydrogen sensors encoded by treponemes from the H(_2_)-rich termite gut. Microb. Ecol. 63, 282–294. doi: 10.1007/s00248-011-9922-8, PMID: 21811792

[ref6] Bauer E. Lampert N. Mikaelyan A. Köhler T. Maekawa K. Brune A. (2015). Physicochemical conditions, metabolites and community structure of the bacterial microbiota in the gut of wood-feeding cockroaches (Blaberidae: Panesthiinae). FEMS Microbiol. Ecol. 91, 1–14. doi: 10.1093/femsec/fiu028, PMID: 25764554

[ref7] Benjamino J. Lincoln S. Srivastava R. Graf J. (2018). Low-abundant bacteria drive compositional changes in the gut microbiota after dietary alteration. Microbiome 6:86. doi: 10.1186/s40168-018-0469-5, PMID: 29747692 PMC5944116

[ref8] Bignell D. E. Roisin Y. Lo N. (2011). Biology of termites: A modern synthesis. 2nd Edn. New York: Springer Publishers, 1–592.

[ref9] Bokulich N. A. Subramanian S. Faith J. J. Gevers D. Gordon J. I. Knight R.. (2013). Quality-filtering vastly improves diversity estimates from Illumina amplicon sequencing. Nat. Methods 10, 57–59. doi: 10.1038/nmeth.2276, PMID: 23202435 PMC3531572

[ref10] Bourguignon T. Lo N. Dietrich C. Šobotník J. Sidek S. Roisin Y.. (2018). Rampant host switching shaped the termite gut microbiome. Curr. Biol. 28, 649–54.e2. doi: 10.1016/j.cub.2018.01.035, PMID: 29429621

[ref11] Bray J. R. Curtis J. T. (1957). An ordination of the upland forest communities of southern Wisconsin. Ecol. Monogr. 27, 325–349. doi: 10.2307/1942268

[ref12] Brennan Y. Callen W. N. Christoffersen L. Dupree P. Goubet F. Healey S.. (2004). Unusual microbial xylanases from insect guts. Appl. Environ. Microbiol. 70, 3609–3617. doi: 10.1128/aem.70.6.3609-3617.2004, PMID: 15184164 PMC427792

[ref13] Brune A. (2014). Symbiotic digestion of lignocellulose in termite guts. Microbiology 12, 168–180. doi: 10.1038/nrmicro3182, PMID: 24487819

[ref14] Brune A. Dietrich C. (2015). The gut microbiota of termites: digesting the dversity in the light of ecology and evolution. Ann. Rev. Microbiol. 69, 145–166. doi: 10.1146/annurev-micro-092412-155715, PMID: 26195303

[ref15] Caporaso J. G. Kuczynski J. Stombaugh J. Bittinger K. Bushman F. D. Costello E. K.. (2010). QIIME allows analysis of high-throughput community sequencing data. Nat. Methods 7, 335–336. doi: 10.1038/nmeth.f.303, PMID: 20383131 PMC3156573

[ref16] Chao A. (1984). Nonparametric estimation of the number of classes in a population. Scanad. J. Stat. 11, 265–270.

[ref17] Chao A. Shen T. J. (2003). Nonparametric estimation of Shannon’s index of diversity when there are unseen species in sample. Environ. Ecol. Stat. 10, 429–443. doi: 10.1023/A:1026096204727

[ref19] Chen S. Zhou Y. Chen Y. Gu J. (2018). FASTP: an ultra-fast all-in-one FASTQ preprocessor. Bioinformatics 34, i884–i890. doi: 10.1093/bioinformatics/bty560, PMID: 30423086 PMC6129281

[ref20] Chouvenc T. Li H. F. Austin J. Bordereau C. Bourguignon T. Cameron S. L.. (2016). Revisiting *Coptotermes* (Isoptera: Rhinotermitidae): a global taxonomic road map for species validity and distribution of an economically important subterranean termite genus. Syst. Entomol. 41, 299–306. doi: 10.1111/syen.12157

[ref21] Colston T. J. Jackson C. R. (2016). Microbiome evolution along divergent branches of the vertebrate tree of life: what is known and unknown. Mol. Ecol. 25, 3776–3800. doi: 10.1111/mec.13730, PMID: 27297628

[ref22] Coy M. Salem T. Denton J. Kovaleva E. Liu Z. Barber D.. (2010). Phenol-oxidizing laccases from the termite gut. Insect Biochem. Mol. Biol. 40, 723–732. doi: 10.1016/j.ibmb.2010.07.004 20691784

[ref23] Dar M. A. Dhole N. P. Xie R. R. Pawar K. D. Ullah K. Rahi P.. (2021b). Valorization potential of a novel bacterial strain, *Bacillus altitudinis* RSP75, towards lignocellulose bioconversion: an assessment of symbiotic bacteria from the stored grain pest. Microorganisms 9:1952. doi: 10.3390/microorganisms9091952, PMID: 34576846 PMC8468446

[ref24] Dar M. A. Shaikh A. F. Pawar K. D. Xie R. R. Sun J. Z. Kandasamy S.. (2021a). Evaluation of cellulose degrading bacteria isolated from the gut system of cotton bollworm, *Helicoverpa armigera* and their potential values in biomass conversion. Peer J. 9:e11254. doi: 10.7717/peerj.11254 PMC11563024 39544200

[ref25] Dar M. A. Xie R. Pandit R. S. Danso B. Dong C. Sun J. (2022). Exploring the region-wise diversity and functions of symbiotic bacteria in the gut system of wood-feeding termite, *Coptotermes formosanus*, toward the degradation of cellulose, hemicellulose, and organic dyes. Insect Sci. 29, 1414–1432. doi: 10.1111/1744-7917.13012, PMID: 35134272

[ref26] Dar M. A. Xie R. Zabed H. M. Ali S. Zhu D. Sun J. (2024). Termite microbial symbiosis as a model for innovative design of lignocellulosic future biorefinery: current paradigms and future perspectives. Biomass 4, 180–201. doi: 10.3390/biomass4010009

[ref27] David L. A. Maurice C. F. Carmody R. N. Gootenberg D. B. Button J. E. Wolfe B. E.. (2014). Diet rapidly and reproducibly alters the human gut microbiome. Nature 505, 559–563. doi: 10.1038/nature12820, PMID: 24336217 PMC3957428

[ref28] Desai M. S. Brune A. (2012). Bacteroidales ectosymbionts of gut flagellates shape the nitrogen-fixing community in dry-wood termites. ISME J. 6, 1302–1313. doi: 10.1038/ismej.2011.194, PMID: 22189498 PMC3379631

[ref30] Dietrich C. Kohler T. Brune A. (2014). The cockroach origin of the termite gut microbiota: patterns in bacterial community structure reflect major evolutionary events. Appl. Environ. Microbiol. 80, 2261–2269. doi: 10.1128/AEM.04206-13, PMID: 24487532 PMC3993134

[ref31] Edgar R. C. (2013). UPARSE: highly accurate OTU sequences from microbial amplicon reads. Nat. Methods 10, 996–998. doi: 10.1038/nmeth.2604, PMID: 23955772

[ref32] Edgar R. C. Haas B. J. Clemente J. C. Quince C. Knight R. (2011). UCHIME improves sensitivity and speed of chimera detection. Bioinformatics 27, 2194–2200. doi: 10.1093/bioinformatics/btr381, PMID: 21700674 PMC3150044

[ref33] Engel P. Moran N. A. (2013). The gut microbiota of insects diversity in structure and function. FEMS Microbiol. Rev. 37, 699–735. doi: 10.1111/1574-6976.12025 23692388

[ref35] Geng A. Cheng Y. Wang Y. Zhu D. Le Y. Wu J.. (2018b). Transcriptome analysis of the digestive system of a wood-feeding termite (*Coptotermes formosanus*) revealed a unique mechanism for effective biomass degradation. Biotechnol. Biofuels 11:24. doi: 10.1186/s13068-018-1015-1, PMID: 29434667 PMC5797411

[ref36] Geng A. Wu J. Xie R. R. Li X. Chang F. X. Sun J. Z. (2018a). Characterization of a laccase from a wood-feeding termite. Entomol. Sin. 25, 251–258. doi: 10.1111/1744-7917.12415, PMID: 27800659

[ref37] Gerber E. Bernard R. Castang S. Chabot N. Coze F. Dreux-Zigha A.. (2015). *Deinococcus* as new chassis for industrial biotechnology: biology, physiology and tools. J. Appl. Microbiol. 119, 1–10. doi: 10.1111/jam.12808, PMID: 25809882 PMC4682472

[ref38] Ghanbari M. Kneifel W. Domig K. J. (2015). A new view of the fish gut microbiome: advances from next-generation sequencing. Aquaculture 448, 464–475. doi: 10.1016/j.aquaculture.2015.06.033

[ref39] Good I. J. (1953). The population frequencies of species and the estimation of population parameters. Biometrika 40, 237–264. doi: 10.1093/biomet/40.3-4.237

[ref40] Guimaraes H. I. P. Santana R. H. Silveira R. Pinto O. H. B. Quirino B. F. Barreto C. C.. (2020). Seasonal variations in soil microbiota profile of termite (*Syntermes wheeleri*) mounds in the Brazilian tropical savanna. Microorganisms 8:1482. doi: 10.3390/microorganisms8101482, PMID: 32992494 PMC7600031

[ref41] Guo M. Wu F. Hao G. Qi Q. Li R. Li N.. (2017). *Bacillus subtilis* improves immunity and disease resistance in rabbits. Front. Immunol. 8:354. doi: 10.3389/fimmu.2017.00354, PMID: 28424690 PMC5372816

[ref42] Hamilton N. E. Ferry M. (2018). Ggtern: ternary diagrams using ggplot 2. J. Stat. Softw. 87, 1–17. doi: 10.18637/jss.v087.c03

[ref44] He S. Ivanova N. Kirton E. Allgaier M. Bergin C. Scheffrahn R. H.. (2013). Comparative metagenomic and metatranscriptomic analysis of hindgut paunch microbiota in wood-and dung-feeding higher termites. PLoS One 8:e61126. doi: 10.1371/journal.pone.0061126, PMID: 23593407 PMC3625147

[ref45] Hemme D. Foucaud-Scheunemann C. (2004). *Leuconostoc*, characteristics, use in dairy technology and prospects in functional foods. Int. Dairy J. 14, 467–494. doi: 10.1016/j.idairyj.2003.10.005

[ref46] Hongoh Y. (2011). Toward the functional analysis of uncultivable, symbiotic microorganisms in the termite gut. Cell. Mol. Life Sci. 68, 1311–1325. doi: 10.1007/s00018-011-0648-z, PMID: 21365277 PMC11114660

[ref47] Hongoh Y. Ekpornprasit L. Inoue T. Moriya S. Trakulnaleamsai S. Ohkuma M.. (2006). Intracolony variation of bacterial gut microbiota among castes and ages in the fungus-growing termite *Macrotermes gilvus*. Mol. Ecol. Resour. 15, 505–516. doi: 10.1111/j.1365-294x.2005.02795.x, PMID: 16448416

[ref49] Hongoh Y. Sato T. Dolan M. F. Noda S. Ui S. Kudo T.. (2007b). The motility symbiont of the termite gut flagellate *Caduceia versatilis* is a member of the “Synergistes” group. Appl. Environ. Microbiol. 73, 6270–6276. doi: 10.1128/AEM.00750-07, PMID: 17675420 PMC2074993

[ref50] Hongoh Y. Sato T. Noda S. Ui S. Kudo T. Ohkuma M. (2007a). *Candidatus* Symbiothrix dinenymphae: bristle-like Bacteroidales ectosymbionts of termite gut protists. Environ. Microbiol. 9, 2631–2635. doi: 10.1111/j.1462-2920.2007.01365.x, PMID: 17803785

[ref51] Hongoh Y. Sharma V. K. Prakash T. Noda S. Taylor T. D. Kudo T.. (2008a). Complete genome of the uncultured termite group 1 bacteria in a single host protist cell. PNAS 105, 5555–5560. doi: 10.1073/pnas.0801389105, PMID: 18391199 PMC2291132

[ref52] Hongoh Y. Sharma V. K. Prakash T. Noda S. Toh H. Taylor T. D.. (2008b). Genome of an endosymbiont coupling N_2_ fixation to cellulolysis within protist cells in termite gut. Science 322, 1108–1109. doi: 10.1126/science.1165578, PMID: 19008447

[ref54] Huang S. Sheng P. Zhang H. (2012). Isolation and identification of cellulolytic bacteria from the gut of *Holotrichia parallela* larvae (Coleoptera: Scarabaeidae). Int. J. Mol. Sci. 13, 2563–2577. doi: 10.3390/ijms13032563, PMID: 22489111 PMC3317674

[ref55] Huang J. Weng L. Zhang X. Long K. An X. Bao J.. (2022). *Trypoxylus dichotomus* gut bacteria provides an effective system for bamboo lignocellulose degradation. Microbiol. Spectr. 10, e02147–e02122. doi: 10.1128/spectrum.02147-22, PMID: 35993784 PMC9602259

[ref56] Husseneder C. Ho H. Y. Blackwell M. (2010). Comparison of the bacterial symbiont composition of the formosan subterranean termite from its native and introduced range. Open Microbiol. J. 4, 53–66. doi: 10.2174/1874285801004010053, PMID: 21347207 PMC3040989

[ref57] Kappler A. Brune A. (2002). Dynamics of redox potential and changes in redox state of iron and humic acids during gut passage in soil-feeding termites (*Cubitermes* spp.). Soil Biol. Biochem. 34, 221–227. doi: 10.1016/S0038-0717(01)00176-6

[ref58] Kemp P. F. Aller J. Y. (2004). Bacterial diversity in aquatic and other environments: what 16S rDNA libraries can tell us. FEMS Microbiol. Ecol. 47, 161–177. doi: 10.1016/s0168-6496(03)00257-5, PMID: 19712332

[ref002] Köhler T. Stingl U. Meuser K. Brune A. (2008). Novel lineages of Planctomycetes densely colonize the alkaline gut of soil-feeding termites (*Cubitermes* spp.). Environ Microbiol. 10, 1260–70. doi: 10.1111/j.1462-2920.2007.01540.x, PMID: 18279348

[ref59] Kohler T. (2011). Diversity and evolutionary patterns in the bacterial gut microbiota of termites and cockroaches. Thesis, University of Marburg, Marburg, Germany.

[ref60] Köhler T. Dietrich C. Scheffrahn R. H. Brune A. (2012). High-resolution analysis of gut environment and bacterial microbiota reveals functional compartmentation of the gut in wood-feeding higher termites (*Nasutitermes* spp.). Appl. Environ. Microbiol. 78, 4691–4701. doi: 10.1128/AEM.00683-12, PMID: 22544239 PMC3370480

[ref61] Lampert N. Mikaelyan A. Brune A. (2019). Diet is not the primary driver of bacterial community structure in the gut of litter feeding cockroaches. BMC Microbiol. 19:238. doi: 10.1186/s12866-019-1601-9, PMID: 31666028 PMC6864750

[ref62] Lax A. R. Osbrink W. L. (2003). United States Department of Agriculture-Agriculture Research Service research on targeted management of the Formosan subterranean termite *Coptotermes formosanus* Shiraki (Isoptera: Rhinotermitidae). Pest Manag. Sci. 59, 788–800. doi: 10.1002/ps.721, PMID: 12846330

[ref63] Lazuka A. Lucas A. O’Donohue M. Hernandez-Raquet G. (2018). Anaerobic lignocellulolytic microbial consortium derived from termite gut: enrichment, lignocellulose degradation and community dynamics. Biotechnol. Biofuels 11:284. doi: 10.1186/s13068-018-1282-x, PMID: 30356893 PMC6191919

[ref64] Lee Y. C. (2002). Control of foraging colonies of subterranean termites, *Coptotermes travians* (lsoptera: Rhinotermitidae) in Malaysia using hexaflumuron baits. Sociobiology 39, 411–416.

[ref65] Lee S. B. Tong R. L. Kim S. H. Im I. G. Su N. Y. (2021). Potential pest status of the formosan subterranean termite, *Coptotermes formosanus* Shiraki (Blattodea: Isoptera: Rhinotermitidae), in response to climate change in the Korean peninsula. Fla. Entomol. 103, 431–437. doi: 10.1653/024.103.00403

[ref67] Lilburn T. G. Kim K. S. Ostrom N. E. Byzek K. R. Leadbetter J. R. Breznak J. A. (2001). Nitrogen fixation by symbiotic and free-living spirochetes. Science 292, 2495–2498. doi: 10.1126/science.1060281, PMID: 11431569

[ref68] Liu N. Li H. Chevrette M. G. Zhang L. Cao L. Zhou H.. (2019). Functional metagenomics reveals abundant polysaccharide degrading gene clusters and cellobiose utilization pathways within gut microbiota of a wood-feeding higher termite. ISME J. 13, 104–117. doi: 10.1038/s41396-018-0255-1, PMID: 30116044 PMC6298952

[ref69] Liu N. Zhang L. Zhou H. Zhang M. Yan X. Wang Q.. (2013). Metagenomic insights into metabolic capacities of the gut microbiota in a fungus-cultivating termite (*Odontotermes yunnanensis*). PLoS One 8:e69184. doi: 10.1371/journal.pone.0069184, PMID: 23874908 PMC3714238

[ref70] Louca S. Parfrey L. W. Doebeli M. (2016). Decoupling function and taxonomy in the global ocean microbiome. Science 353, 1272–1277. doi: 10.1126/science.aaf4507, PMID: 27634532

[ref72] Magoč T. Salzberg S. L. (2011). FLASH: fast length adjustment of short reads to improve genome assemblies. Bioinformatics 27, 2957–2963. doi: 10.1093/bioinformatics/btr507, PMID: 21903629 PMC3198573

[ref73] Makonde H. M. Boga H. I. Osiemo Z. Mwirichia R. Mackenzie L. M. Goker M.. (2013). 16S-rRNA-based analysis of bacterial diversity in the gut of fungus-cultivating termites (*Microtermes* and *Odontotermes* species). Antonie Van Leeuwenhoek 104, 869–883. doi: 10.1007/s10482-013-0001-7, PMID: 23942613

[ref74] Marynowska M. Goux X. Sillam-Dussès D. Rouland-Lefèvre C. Halder R. Wilmes P.. (2020). Compositional and functional characterisation of biomass-degrading microbial communities in guts of plant fibre- and soil-feeding higher termites. Microbiome 8:96. doi: 10.1186/s40168-020-00872-3, PMID: 32576253 PMC7313118

[ref75] Méheust R. Castelle C. J. Matheus Carnevali P. B. Farag I. F. He C. Chen L. X.. (2020). Groundwater *Elusimicrobia* are metabolically diverse compared to gut microbiome *Elusimicrobia* and some have a novel nitrogenase paralog. ISME J. 14, 2907–2922. doi: 10.1038/s41396-020-0716-1, PMID: 32681159 PMC7785019

[ref76] Mikaelyan A. Dietrich C. Köhler T. Poulsen M. Sillam-Dussès D. Brune A. (2015). Diet is the primary determinant of bacterial community structure in the guts of higher termites. Mol. Ecol. 24, 5284–5295. doi: 10.1111/mec.13376, PMID: 26348261

[ref77] Mikaelyan A. Meuser K. Brune A. (2017). Microenvironmental heterogeneity of gut compartments drives bacterial community structure in wood- and humus feeding higher termites. FEMS Microbiol. Ecol. 93, 1–11. doi: 10.1093/femsec/fiw210, PMID: 27798065

[ref78] Mitaka Y. Vargo E. L. (2023). Media made from brown-rotted elm and pine wood for rearing *Reticulitermes* termites. Insect. Soc. 70, 381–389. doi: 10.1007/s00040-023-00928-1

[ref80] Mroz Z. Reese D. E. Øverland M. Van Diepen J. T. M. Kogut J. (2002). The effects of potassium diformate and its molecular constituents on the apparent ileal and fecal digestibility and retention of nutrients in growing-finishing pigs. J. Anim. Sci. 80, 681–690. doi: 10.2527/2002.803681x, PMID: 11892679

[ref81] Nakajima H. Hongoh Y. Noda S. Yoshida Y. Usami R. Kudo T.. (2006). Phylogenetic and morphological diversity of Bacteroidales members associated with the gut wall of termites. Biosci. Biotechnol. Biochem. 70, 211–218. doi: 10.1271/bbb.70.211, PMID: 16428839

[ref82] Nalepa C. A. (2020). Origin of mutualism between termites and flagellated gut protists: transition from horizontal to vertical transmission. Front. Ecol. Evol. 8:14. doi: 10.3389/fevo.2020.00014

[ref83] Ngugi D. K. Brune A. (2012). Nitrate reduction, nitrous oxide formation, and anaerobic ammonia oxidation to nitrite in the gut of soil-feeding termites (*Cubitermes* and *Ophiotermes* spp). Environ. Microbiol. 14, 860–871. doi: 10.1111/j.1462-2920.2011.02648.x, PMID: 22118414

[ref85] Noda S. Iida T. Kitade O. Nakajima H. Kudo T. Ohkuma M. (2005). Endosymbiotic Bacteroidales bacteria of the flagellated protist Pseudotrichonympha grassii in the gut of the termite *Coptotermes formosanus*. Appl. Environ. Microbiol. 71, 8811–8817. doi: 10.1128/AEM.71.12.8811-8817.2005, PMID: 16332877 PMC1317455

[ref86] Noda S. Kitade O. Inoue T. Kawai M. Kanuka M. Hiroshima K.. (2007). Cospeciation in the triplex symbiosis of termite gut protists (*Pseudotrichonympha* spp.), their hosts, and their bacterial endosymbionts. Mol. Ecol. 16, 1257–1266. doi: 10.1111/j.1365-294x.2006.03219.x, PMID: 17391411

[ref87] Oksanen J. Blanchet F. G. Kindt R. Legendre P. Minchin P. R. O'Hara R. B.. (2010). Vegan: community ecology package. R package version 1.17-4. Available at: http://cran.r-project.org (Accessed 23, 2010).

[ref88] Pan X. Raaijmakers J. M. Carrion V. J. (2023). Importance of Bacteroidetes in host-microbe interactions and ecosystem functioning. Trends Microbiol. 31, 959–971. doi: 10.1016/j.tim.2023.03.018, PMID: 37173204

[ref89] Parada A. E. Needham D. M. Fuhrman J. A. (2016). Every base matters: assessing small subunit rRNA primers for marine microbiomes with mock communities, time series and global field samples. Environ. Microbiol. 18, 1403–1414. doi: 10.1111/1462-2920.13023, PMID: 26271760

[ref90] R Core Team (2023). R: A language and environment for statistical computing. R Foundation for Statistical Computing. Vienna, Austria. Available at: https://www.R-project.org/.

[ref91] Rahman N. A. Parks D. H. Willner D. L. Engelbrektson A. L. Goffredi S. K. Warnecke F.. (2015). A molecular survey of Australian and north American termite genera indicates that vertical inheritance is the primary force shaping termite gut microbiomes. Microbiome 3, 5–16. doi: 10.1186/s40168-015-0067-8, PMID: 25830022 PMC4379614

[ref92] Ramette A. (2007). Multivariate analyses in microbial ecology. FEMS Microbiol. Ecol. 62, 142–160. doi: 10.1111/j.1574-6941.2007.00375.x, PMID: 17892477 PMC2121141

[ref001] Reid N. M. Addison S. L. West M. A. (2014). The bacterial microbiota of *Stolotermes ruficeps* (Stolotermitidae), a phylogenetically basal termite endemic to New Zealand. FEMS Microbiol. Ecol. 90, 678–688. doi: 10.1111/1574-6941.12424 25196080

[ref93] Rust M. K. Su S. Y. (2012). Managing social insects of urban importance. Annu. Rev. Entomol. 57, 355–375. doi: 10.1146/annurev-ento-120710-100634 21942844

[ref94] Sato T. Hongoh Y. Noda S. Hattori S. Ui S. Ohkuma M. (2009). *Candidatus* Desulfovibrio trichonymphae, a novel intracellular symbiont of the flagellate Trichonympha agilis in termite gut. Environ. Microbiol. 11, 1007–1015. doi: 10.1111/j.1462-2920.2008.01827.x, PMID: 19170725

[ref95] Sato T. Kuwahara H. Fujita K. Noda S. Kihara K. Yamada A.. (2014). Intranuclear verrucomicrobial symbionts and evidence of lateral gene transfer to the host protist in the termite gut. ISME J. 8, 1008–1019. doi: 10.1038/ismej.2013.222, PMID: 24335826 PMC3996698

[ref96] Scharf M. E. (2015a). Omic research in termites: an overview and a roadmap. Front. Genet. 6:76. doi: 10.3389/fgene.2015.00076, PMID: 25821456 PMC4358217

[ref97] Scharf M. E. (2015b). Termites as targets and models for biotechnology. Annu. Rev. Entomol. 60, 77–102. doi: 10.1146/annurev-ento-010814-020902, PMID: 25341102

[ref98] Scharf M. E. Peterson B. F. (2021). A century of synergy in termite Symbiosis research: linking the past with new genomic insights. Annu. Rev. Entomol. 66, 23–43. doi: 10.1146/annurev-ento-022420-074746, PMID: 33417825

[ref99] Segata N. Izard J. Waldron L. Gevers D. Miropolsky L. Garrett W. S.. (2011). Metagenomic biomarker discovery and explanation. Genome Biol. 12:R60. doi: 10.1186/gb-2011-12-6-r60, PMID: 21702898 PMC3218848

[ref100] Shinzato N. Muramatsu M. Matsui T. Watanabe Y. (2005). Molecular phylogenetic diversity of the bacterial community in the gut of the termite *Coptotermes formosanus*. Biosci. Biotechnol. Biochem. 69, 1145–1155. doi: 10.1271/bbb.69.1145, PMID: 15973046

[ref101] Smith C. C. Srygley R. B. Healy F. Swaminath K. Mueller U. G. (2017). Spatial structure of the Mormon cricket gut microbiome and its predicted contribution to nutrition and immune function. Front. Microbiol. 8:801. doi: 10.3389/fmicb.2017.00801, PMID: 28553263 PMC5427142

[ref103] Stingl U. Radek R. (2005). *Endomicrobia*: cytoplasmic symbionts of termite gut protozoa form a separate phylum of prokaryotes. Appl. Environ. Microbiol. 71, 1473–1479. doi: 10.1128/AEM.71.3.1473-1479.2005, PMID: 15746350 PMC1065190

[ref104] Su N. Y. (2003). Overview of the global distribution and control of the Formosan subterranean termite. Sociobiology 41, 7–16.

[ref106] Sun J. Ding S. Y. Peterson J. D. (2014). “Biomass and its biorefinery: novel approaches from nature-inspired strategies and technology” in Biological conversion of biomass for fuels and chemicals: Explorations from natural utilization systems. eds. Sun J. Ding S. Y. Peterson J. D. (United States of America: The Royal Society of Chemistry), 1–13.

[ref107] Sun M. Wang Q. Zhang M. M. Zhang G. H. Wu T. Liu R.. (2020). *Leuconostoc pseudomesenteroides* improves microbiota dysbiosis and liver metabolism imbalance and ameliorates the correlation between dihydroceramide and strains of Firmicutes and Proteobacteria in high fat diet obese mice. Food Function 11, 6855–6865. doi: 10.1039/d0fo01009j, PMID: 32666978

[ref108] Sybesma W. Starrenburg M. Tijsseling L. Hoefnagel M. H. N. Hugenholtz J. (2003). Effect of cultivation conditions on folate production by lactic bacteria. Appl. Environ. Microbiol. 69, 4542–4548. doi: 10.1128/aem.69.8.4542-4548.2003, PMID: 12902240 PMC169137

[ref109] Tholen A. Brune A. (2000). Impact of oxygen on metabolic fluxes and *in situ* rates of reductive acetogenesis in the hindgut of the wood-feeding termite *Reticulitermes flavipes*. Environ. Microbiol. 2, 436–449. doi: 10.1046/j.1462-2920.2000.00127.x, PMID: 11234932

[ref111] Tokuda G. (2021). Origin of symbiotic gut spirochetes as key players in the nutrition of termites. Environ. Microbiol. 23, 4092–4097. doi: 10.1111/1462-2920.15625, PMID: 34097340

[ref112] Tokuda G. Lo N. Watanabe H. (2005). Marked variations in patterns of cellulase activity against crystalline- vs carboxymethyl-cellulose in the digestive systems of diverse, wood-feeding termites. Physiol. Entomol. 30, 372–380. doi: 10.1111/j.1365-3032.2005.00473.x

[ref113] Tokuda G. Mikaelyan A. Fukui C. Matsuura Y. Watanabe H. Fujishima M.. (2018). Fiber-associated spirochetes are major agents of hemicellulose degradation in the hindgut of wood-feeding higher termites. PNAS 115, E11996–E12004. doi: 10.1073/pnas.1810550115, PMID: 30504145 PMC6304966

[ref114] Tytgat H. L. P. Nobrega F. L. van der Oost J. de Vos W. M. (2019). Bowel biofilms: tipping points between a healthy and compromised gut? Trends Microbiol. 27, 17–25. doi: 10.1016/j.tim.2018.08.009 30219265

[ref115] Utami Y. D. Kuwahara H. Murakami T. Morikawa T. Sugaya K. Kihara K.. (2018). Phylogenetic diversity and single-cell genome analysis of “Melainabacteria”, a non-photosynthetic cyanobacterial group, in the termite gut. Microbes Environ. 33, 50–57. doi: 10.1264/jsme2.ME17137, PMID: 29415909 PMC5877343

[ref116] Visser A. A. Nobre T. Currie C. R. Aanen D. K. Poulsen M. (2012). Exploring the potential for actinobacteria as defensive symbionts in fungus-growing termites. Microb. Ecol. 63, 975–985. doi: 10.1007/s00248-011-9987-4, PMID: 22173371

[ref117] Wang Q. Garrity G. M. Tiedje J. M. Cole J. R. (2007). Naive Bayesian classifier for rapid assignment of rRNA sequences into the new bacterial taxonomy. Appl. Environ. Microbiol. 73, 5261–5267. doi: 10.1128/aem.00062-07, PMID: 17586664 PMC1950982

[ref118] Wang C. Powell J. E. Liu Y. (2002). A literature review of the biology and ecology of *Coptotermes formosanus* (Isoptera: Rhinotermitidae) in China. Sociobiology 40, 343–364.

[ref119] Ward T. Larson J. Meulemans J. Hillmann B. Lynch J. Sidiropoulos D.. (2017). Bug base predicts organism level microbiome phenotypes. Bio Rxiv 2017. doi: 10.1101/133462

[ref120] Ware J. L. Grimaldi D. A. Engel M. S. (2010). The effects of fossil placement and calibration on divergence times and rates: an example from the termites (Insecta: Isoptera). Arthropod Struct. Dev. 39, 204–219. doi: 10.1016/j.asd.2009.11.003, PMID: 19962450

[ref121] Warnecke F. Luginbühl P. Ivanova N. Ghassemian M. Richardson T. H. Stege J. T.. (2007). Metagenomic and functional analysis of hindgut microbiota of a wood-feeding higher termite. Nature 450, 560–565. doi: 10.1038/nature06269, PMID: 18033299

[ref122] Weymarn N. Hujanen M. Leisola M. (2002). Production of D-mannitol by heterofermentatative lactic acid bacteria. Process Biochem. 37, 1207–1213. doi: 10.1016/S0032-9592(01)00339-9

[ref123] Xie R. Chen Chen D. Wang S. Danso B. Dar M. A. Pandit R. S.. (2023). Host-specific diversity of culturable bacteria in the gut systems of fungus-growing termites and their potential functions towards lignocellulose bioconversion. Insects 14:403. doi: 10.3390/insects14040403, PMID: 37103218 PMC10146277

[ref124] Xie S. X. Syrenne R. Sun S. Yuan J. S. (2014). Exploration of natural biomass utilization systems (NBUS) for advanced biofuel-from systems biology to synthetic design. Curr. Opin. Biotechnol. 27, 195–203. doi: 10.1016/j.copbio.2014.02.007, PMID: 24657913

[ref125] Xie L. Zhang L. Zhong Y. Liu N. Long Y. H. Wang S. Y.. (2012). Profiling the metatranscriptome of the protistan community in *Coptotermes formosanus* with emphasis on the lignocellulolytic system. Genomics 99, 246–255. doi: 10.1016/j.ygeno.2012.01.009, PMID: 22326742

[ref126] Yamada A. Inoue T. Noda Y. Hongoh H. Ohkuma M. (2007). Evolutionary trend of phylogenetic diversity of nitrogen fixation genes in the gut community of wood-feeding termites. Mol. Ecol. 16, 3768–3777. doi: 10.1111/j.1365-294x.2007.03326.x, PMID: 17850544

[ref127] Yang H. Schmitt-Wagner D. Stingl U. Brune A. (2005). Niche heterogeneity determines bacterial community structure in the termite gut (*Reticulitermes santonensis*). Environ. Microbiol. 7, 916–932. doi: 10.1111/j.1462-2920.2005.00760.x, PMID: 15946289

[ref128] Zhang S. Song F. Wang J. Xu L. Zhang Y. Zhou W.. (2024). Gut microbiota facilitate adaptation of invasive moths to new host plants. ISME J. 18:wrae 031. doi: 10.1093/ismejo/wrae031, PMID: 38423525 PMC10980833

[ref129] Zhang Y. Zhang S. Xu L. (2023). The pivotal roles of gut microbiota in insect plant interactions for sustainable pest management. NPJ Biofilms Microbiomes 9, 66–12. doi: 10.1038/s41522-023-00435-y, PMID: 37735530 PMC10514296

