# Supplementary material for: Elucidating the structure, and composition of bacterial symbionts in the gut regions of wood-feeding termite, Coptotermes formosanus and their functional profile towards lignocellulolytic systems
Source: Front Microbiol. 2024 May 22;15:1395568. doi: 10.3389/fmicb.2024.1395568 (PMC11155305; doi:10.3389/fmicb.2024.1395568)
Supplement: Supplementary file 1 [file Data_Sheet_1.doc]

**Supplementary Information**

**For**

**Elucidating the structure, and composition of bacterial symbionts in the gut regions of wood-feeding termite, Coptotermes formosanus and their functional profile towards lignocellulolytic systems**

**Journal: Frontiers in Microbiology**

Mudasir A. Dar^1^*,*^2$^, Rongrong Xie^1$^, Luohui Jing^1^, Xu Qing^1^, Shahbaz Ali^1^, Radhakrishna S. Pandit^2^, Chaitali M. Shaha^2^, Jianzhong Sun^1^*

^1^Biofuels Institute, School of the Environment and Safety Engineering, Jiangsu University, Zhenjiang-212013, China.

^2^Department of Zoology, Savitribai Phule Pune University, Ganeshkhind, Pune- 411007, India.

***Corresponding authors:** [jzsun1002@ujs.edu.cn](mailto:jzsun1002@ujs.edu.cn) (J.Z.S.); [rrxie@ujs.edu.cn](mailto:rrxie@ujs.edu.cn) (R.X.)

(Tel.: +86 15052919625, fax.: +86 0511-88790955)

^$^Authors contributed equally to this work.

**Running title: Diversity and functions of gut bacteria in *Coptotermes formosanus***


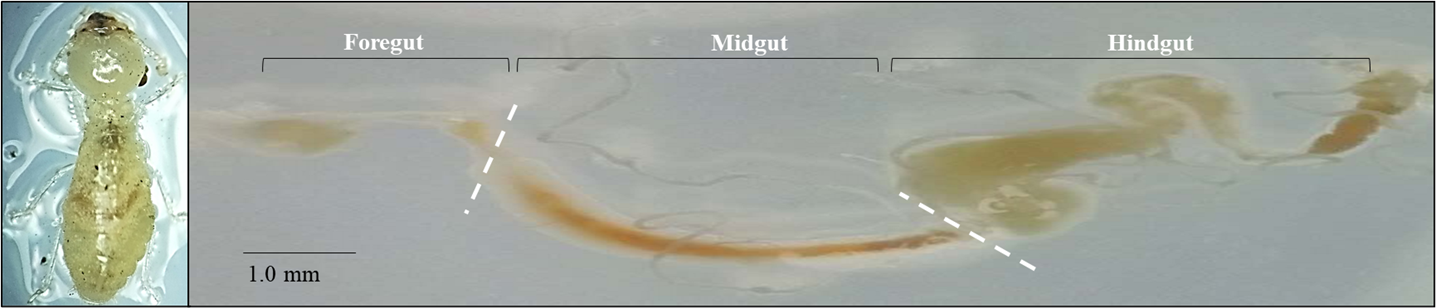


**Fig. S1.** Gut system of the *Coptotermes formosanus* showing foregut, midgut and hindgut regions. The white dashed lines depict the points where the cuts were made to separate the gut regions.


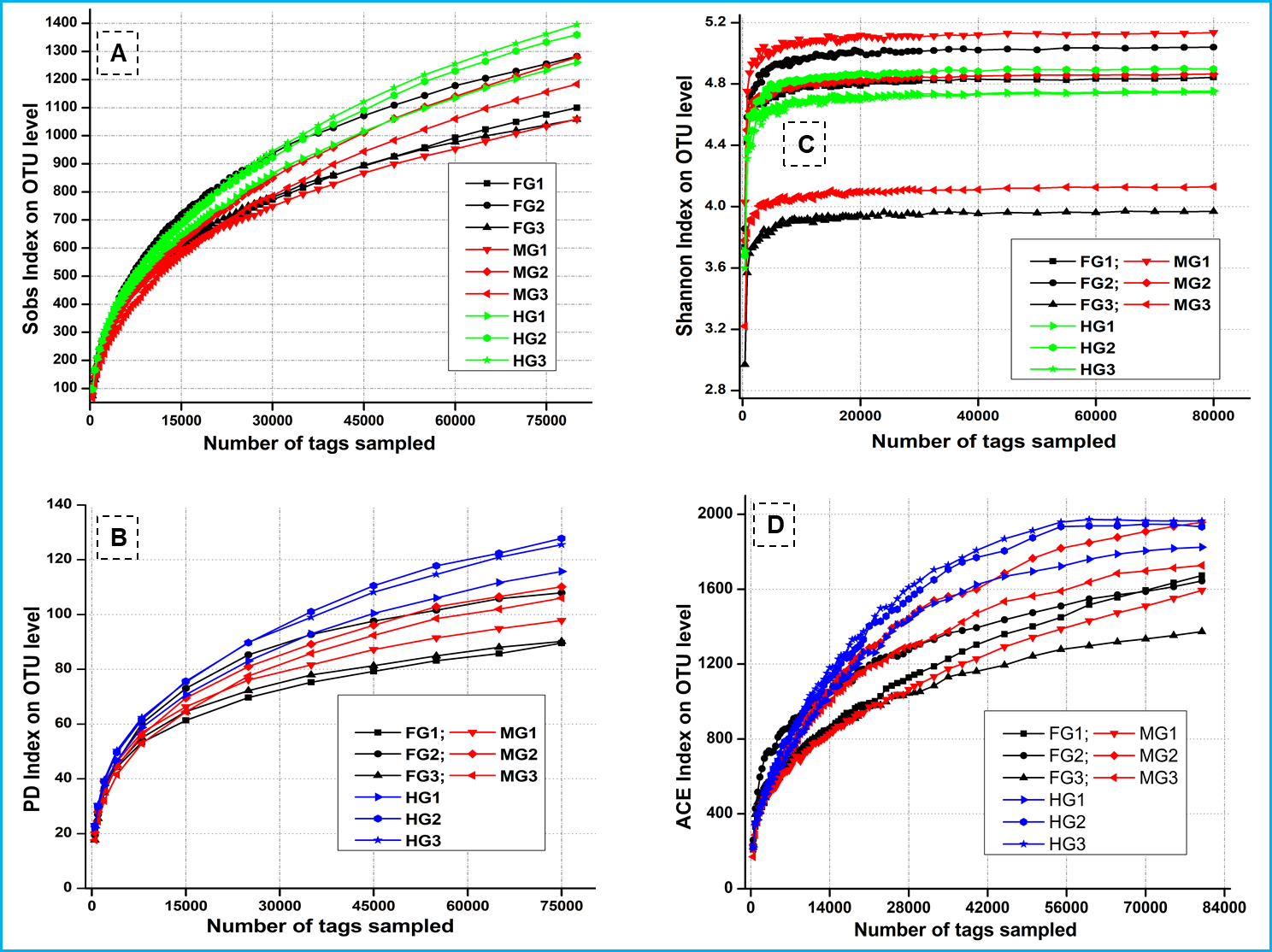


**Figure S2.** Graphical curves demonstrating the comparison of alpha diversity (A Sob index, B: Shannon index, C: phylogenetic diversity index and D: ACE index) of the bacterial community in the gut system of *C. formosanus* between Foregut, midgut and hindgut regions.


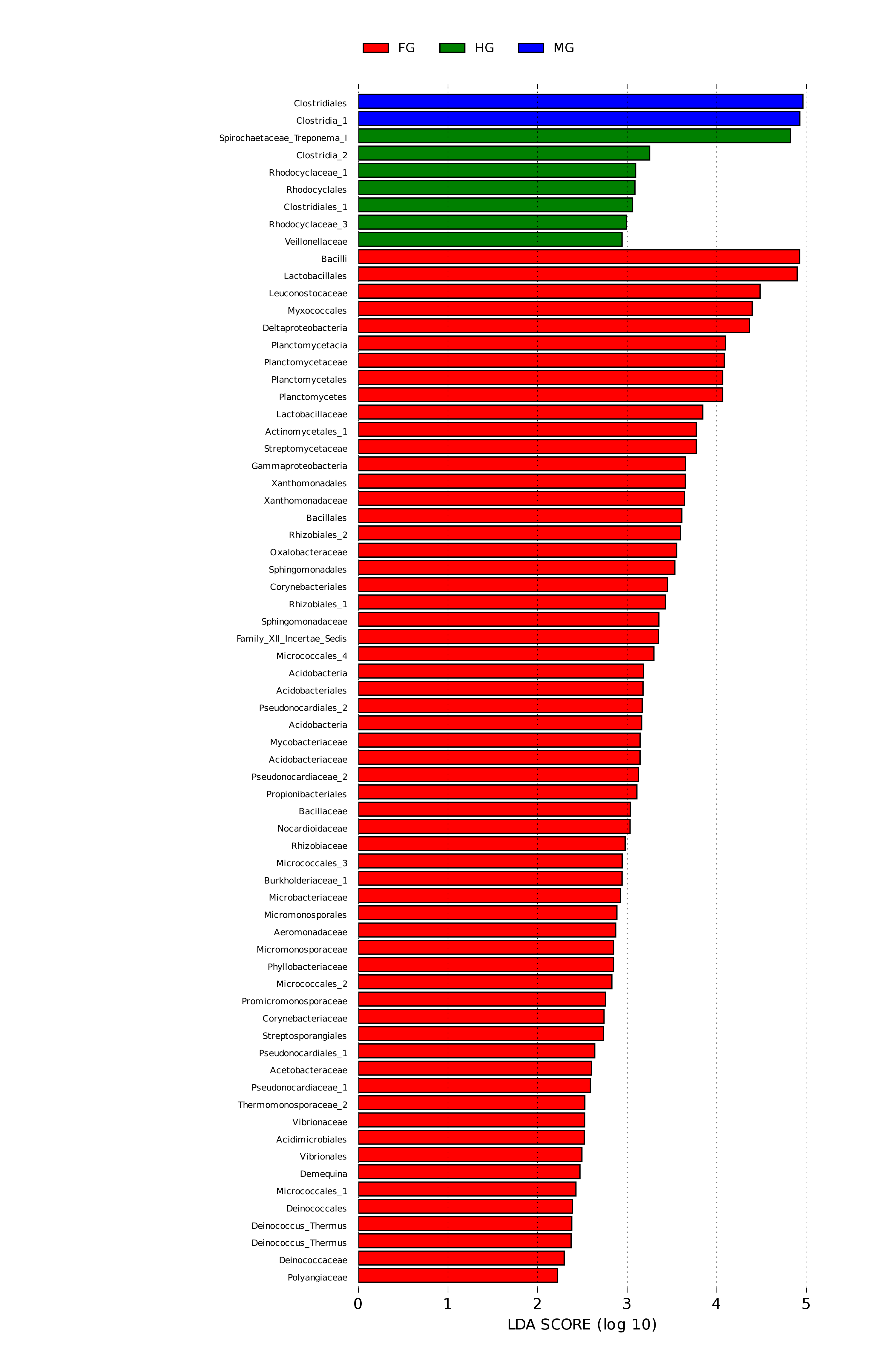


**Figure S3.** LEfSe analysis of the bacterial families in the gut-regions of the wood-feeding termite, *Coptotermes formosanus*.


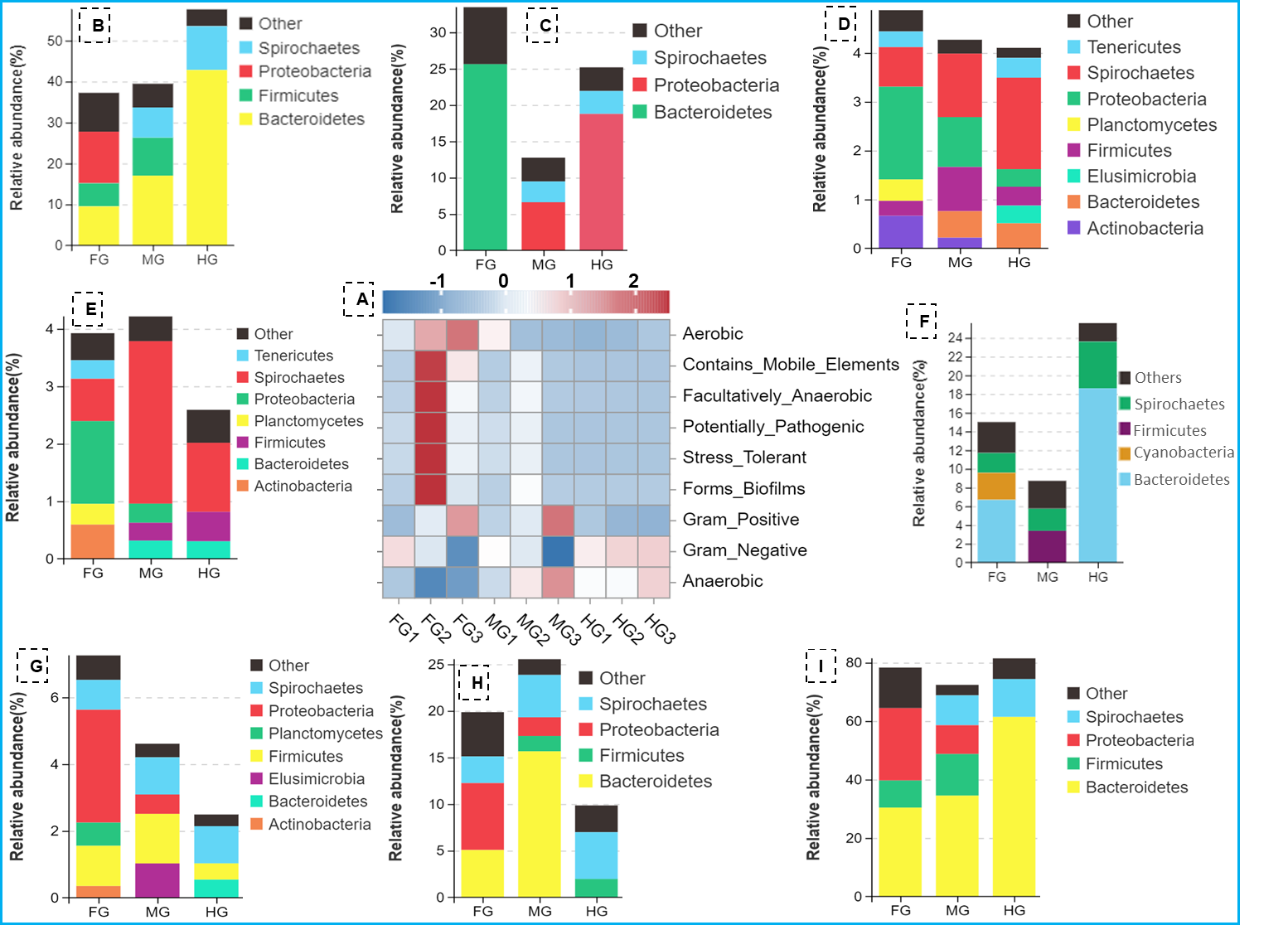


**Figure S4.** A: Function distribution of the bacteria in different regions of the gut system of *C. formosanus*. B-J: Relative abundance and contribution of the major bacterial phyla towards particular function within the gut-regions of the termite. B: Aerobic bacteria, C: Anaerobes, D: Facultatively anaerobic bacteria, E: Biofilm forming bacteria, F: Stress tolerant bacteria, G: Mobile genetic element, H: Gram +, I: Gram-negative

Table S1. Data processing and summery statistics of the metagenomic assembly of reads generated from the bacterial communities in different gut regions of *C. formosanus*.

| **Sample** | **Raw Tags** | **Clean tags** | **Chimera** | **Effective tags** | **Taxon tags** | **Unclassified tags** | **Singleton tags** | **OTUs** | **Average OTUs** | **Gut-region** |
| --- | --- | --- | --- | --- | --- | --- | --- | --- | --- | --- |
| FG1 | 117394 | 115474 | 537 | 114937 | 107470 | 0 | 7467 | 1218 | 1256.6 | **Foregut** |
| FG2 | 121515 | 119215 | 562 | 118653 | 109742 | 0 | 8911 | 1393 |  |  |
| FG3 | 119092 | 116138 | 304 | 115834 | 110091 | 0 | 5743 | 1159 |  |  |
| MG1 | 117471 | 115307 | 887 | 114420 | 106339 | 0 | 8081 | 1165 | 1270.6 |  |
| MG2 | 110276 | 108338 | 1960 | 106378 | 92288 | 0 | 14090 | 1347 |  | **Midgut** |
| MG3 | 121464 | 118720 | 1656 | 117064 | 104649 | 0 | 12415 | 1300 |  |  |
| HG1 | 123411 | 121403 | 4009 | 117394 | 91266 | 0 | 26128 | 1317 | 1379 |  |
| HG2 | 124869 | 122746 | 4523 | 118223 | 88071 | 0 | 30152 | 1406 |  | **Hindgut** |
| HG3 | 117503 | 115628 | 4085 | 111543 | 83119 | 0 | 28424 | 1414 |  |  |
| **Total** | **1072995** | **1052969** | **18503** | **1034446** | **893035** | **0** | **141411** | **11719** |  |  |

OTUs: Operational taxonomic units; FG: foregut; MG: midgut; HG: hindgut
